# Risks and benefits of direct oral anticoagulants versus warfarin in a real world setting: cohort study in primary care

**DOI:** 10.1136/bmj.k2505

**Published:** 2018-07-04

**Authors:** Yana Vinogradova, Carol Coupland, Trevor Hill, Julia Hippisley-Cox

**Affiliations:** 1 Division of Primary Care, 13th floor, Tower Building, University Park, University of Nottingham, Nottingham NG2 7RD, UK

## Abstract

**Objective:**

To investigate the associations between direct oral anticoagulants (DOACs) and risks of bleeding, ischaemic stroke, venous thromboembolism, and all cause mortality compared with warfarin.

**Design:**

Prospective open cohort study.

**Setting:**

UK general practices contributing to QResearch or Clinical Practice Research Datalink.

**Participants:**

132 231 warfarin, 7744 dabigatran, 37 863 rivaroxaban, and 18 223 apixaban users without anticoagulant prescriptions for 12 months before study entry, subgrouped into 103 270 patients with atrial fibrillation and 92 791 without atrial fibrillation between 2011 and 2016.

**Main outcome measures:**

Major bleeding leading to hospital admission or death. Specific sites of bleeding and all cause mortality were also studied.

**Results:**

In patients with atrial fibrillation, compared with warfarin, apixaban was associated with a decreased risk of major bleeding (adjusted hazard ratio 0.66, 95% confidence interval 0.54 to 0.79) and intracranial bleeding (0.40, 0.25 to 0.64); dabigatran was associated with a decreased risk of intracranial bleeding (0.45, 0.26 to 0.77). An increased risk of all cause mortality was observed in patients taking rivaroxaban (1.19, 1.09 to 1.29) or on lower doses of apixaban (1.27, 1.12 to 1.45). In patients without atrial fibrillation, compared with warfarin, apixaban was associated with a decreased risk of major bleeding (0.60, 0.46 to 0.79), any gastrointestinal bleeding (0.55, 0.37 to 0.83), and upper gastrointestinal bleeding (0.55, 0.36 to 0.83); rivaroxaban was associated with a decreased risk of intracranial bleeding (0.54, 0.35 to 0.82). Increased risk of all cause mortality was observed in patients taking rivaroxaban (1.51, 1.38 to 1.66) and those on lower doses of apixaban (1.34, 1.13 to 1.58).

**Conclusions:**

Overall, apixaban was found to be the safest drug, with reduced risks of major, intracranial, and gastrointestinal bleeding compared with warfarin. Rivaroxaban and low dose apixaban were, however, associated with increased risks of all cause mortality compared with warfarin.

## Introduction

Anticoagulants are used for the prevention and treatment of venous thromboembolism and to reduce the risk of stroke in patients with either atrial fibrillation or after acute pulmonary embolism, deep vein thrombosis, or hip or knee replacement surgery.^1^ ^2^ ^3^ ^4^ Warfarin has been used for six decades but in the last eight years its use has been gradually replaced by a new class of direct acting oral anticoagulants (DOACs) including dabigatran, rivaroxaban, and apixaban. Unlike warfarin, these drugs have set doses and do not generally require regular international normalisation ratio blood test monitoring.^5^ They also have faster onset and offset of action. There are, however, some concerns regarding the safety of DOACs with respect to bleeding because there is an absence of or a limited choice of antidotes, some of which are also expensive.^6^ ^7^

Atrial fibrillation is the most common condition requiring anticoagulants, and most clinical trial evidence has been based on this group of patients. These trials have established non-inferiority in the anticoagulating qualities of DOACs compared with warfarin in controlled trial settings,^8^ ^9^ ^10^ but there are residual concerns regarding their safety, particularly in more real world settings, where they are prescribed to a broad range of patients. A recent meta-analysis has shown that apixaban has advantages over warfarin, providing a better balance between efficacy and safety.^11^ The included studies were differently designed, and none provided data for all DOACs. These findings, therefore, represent only indirect comparisons between different types of DOACs, derived using network meta-analysis techniques.

Most well powered observational studies have also focused on patients with atrial fibrillation.^12^ ^13^ ^14^ ^15^ ^16^ ^17^ ^18^ ^19^ ^20^ ^21^ ^22^ ^23^ ^24^ ^25^ Only two have provided data for the wider population,^13^ ^15^ and only one of these presented results for the group without atrial fibrillation.^13^ Both studies were based on data from commercially insured patients, containing billing information, and were conducted a few years ago, when warfarin was more commonly prescribed. Our study aims, for all incident users of anticoagulants, to compare the risks (major bleeding and mortality) and benefits (reduced ischaemic stroke and venous thromboembolism) associated with the three commonest types of DOACs compared with warfarin. We provide separate results for the group with atrial fibrillation and for the group prescribed the drugs because of other conditions.

## Methods

### Data sources

We used two UK primary care databases QResearch and Clinical Practice Research Datalink (CPRD). Each is representative of the national population in terms of the number of practices and of patients that contribute.^26^ Both have been widely validated against other sources of information and used in a wide range of clinical studies.^27^ All 1457 QResearch (version 42) and 357 CPRD (November 2016) practices were linked at the patient level to hospital admissions data, which provided dates and diagnoses for hospital stays.^28^ These practices were also linked to mortality data supplied by the Office for National Statistics, which include diagnoses and dates of death**.** Most patients in linked practices also had information on their level of deprivation based on quintiles of Townsend score and provided by Census 2011.^29^ We used READ codes to extract the information from general practices and ICD-10 (international classification of diseases, 10th revision) codes for Hospital Episode Statistics and Office for National Statistics data (see supplementary table 1).

### Study design

We used a new-user design to capture all events occurring after starting treatment and to reduce the impact of confounding.^30^ For a study period from January 2011 to the latest date of Hospital Episode Statistics linked data (October 2016 for QResearch and March 2016 for CPRD), patients prescribed the oral anticoagulants warfarin, dabigatran, rivaroxaban, and apixaban, and aged from 21 to 99 at study entry date, formed the cohort. The entry date was defined as the date of the first prescription of any of the anticoagulant drugs. To facilitate a direct comparison between new users of direct oral anticoagulants (DOACs) against new users of warfarin, and to reduce the impact of indication bias, patients were excluded if they had any anticoagulant prescription in the last 12 months before the entry date. To ensure the quality of data, patients were also excluded if they had either fewer than 12 months of records before entry or had no valid Townsend score.

Patients were followed from their first prescription of an anticoagulant until they experienced an outcome of interest or were censored. Patients were censored when: they stopped or suspended treatment (at 30 days after the expected end date of any prescription, where the gap between the expected prescription end date and the start date of any subsequent prescription was more than 30 days); they switched treatment (at the day before the prescription start for a different anticoagulant); they left a practice (at the day of deregistration); they died; or the study period ended. For the analyses of dosages, we also censored patients when they changed to a different dose.

### Outcomes

To assess the scale of unintended adverse events of anticoagulant treatment, the primary outcome was major bleeding after entry to the study which led to a hospital admission or death, based on linked hospital or mortality records. The first occurrence was used in the analyses of specific outcomes including intracranial bleed, haematuria, haemoptysis, and gastrointestinal bleed (also separated into upper and lower, where recorded), because these were identified as possibly preventable and potentially life threatening or life changing.

To assess the efficacy of anticoagulant treatments, the following secondary outcomes were considered: ischaemic stroke, venous thromboembolism, and all cause mortality. The outcome date was the earliest record after entry to the study from GP, hospital, and mortality data records. These analyses were focused on primary prevention so patients having venous thromboembolism events before entry to the study were excluded from the analysis of the risk of venous thromboembolism. Similarly, patients with previous ischaemic strokes were excluded from the analysis of the risk of ischaemic stroke.

### Exposure to anticoagulants

Three DOACs – dabigatran, rivaroxaban, and apixaban – were compared with warfarin. Edoxaban was not included because it was licensed in the UK at the end of 2015. Acenocoumarol and phenindione were not included because they have been rarely prescribed in the UK.

Extracted data for anticoagulant prescriptions contained the preparation details, number of days, and number of tablets per day. The daily dose was averaged for each prescription and categorised as lower or higher than the recommended daily dose: 300 mg for dabigatran, 20 mg for rivaroxaban, and 10 mg for apixaban. In the subcohort with atrial fibrillation, higher dose corresponds to standard dose. Precise dosages for warfarin were not available because they vary according to international normalisation ratio measurement and are not consistently recorded in general practice.

### Confounding factors

It is possible that patients at higher risk of bleeding may preferentially be prescribed DOACs rather than warfarin, so all analyses were adjusted for demographic and clinical variables, either because they may have been used as indicators for prescribing a specific anticoagulant or because they have possible associations with increased risk of bleeding, ischaemic stroke, or venous thromboembolism. We similarly adjusted for comorbidities, previous events, and drugs also used as indicators or associated with increased risks.^31^ The covariates were assessed at the date when the anticoagulant was first prescribed.

Demographic and lifestyle variables, included because they affect the risk of bleeding, ischaemic stroke, or venous thromboembolism, were: sex; age at entry to the study;^32^ self assigned ethnicity; smoking status; alcohol use;^33^ and deprivation.^32^ ^34^ Body mass index and systolic blood pressure were included for the same reason.

Comorbidities included if recorded before the drug start were: alcohol dependence; bleeding disorders; cancer (the 12 most commonly occurring types); chronic liver disease or pancreatitis;^33^ chronic obstructive pulmonary disease; chronic renal disease;^33^ congestive cardiac failure; coronary heart disease; diabetes; dyspepsia or heartburn; treated hypertension;^33^ previous ischaemic stroke or transient ischaemic attack; oesophageal varices; peptic ulcer; valvular heart disease; venous thromboembolism; and previous bleed (including intracranial, haematuria, haemoptysis, or gastrointestinal). If recorded in the six months before the start of anticoagulant treatment, falls or hip fractures and hip or knee replacement operations were both included in the analysis.

Recent and concurrent drug use, included in the analysis because they may affect the risk of bleeding or interact with anticoagulants, were: proton pump inhibitors; macrolide antibiotics; antiplatelets;^33^ antidepressants;^35^ anticonvulsants (phenytoin or carbamazepine); non-steroidal anti-inflammatory drugs; corticosteroids; and statins. For women, hormonal treatments, which included hormone replacement therapy and oral contraceptives, were also added to the analysis of venous thromboembolism outcome because they may increase the risk of venous thromboembolism.

Finally, year of entry to the study was included as a confounder both because of changes in recorded rates of outcomes over the study period and because the balance of prescribing between the different anticoagulants was also changing. Specifically, rates of bleeding, ischaemic stroke, and venous thromboembolism were changing in the general population and, while at the beginning of the study warfarin was overwhelmingly the most common anticoagulant prescription, by the end of the study combined prescription rates for DOACs were considerably higher than for warfarin.

### Statistical analysis

The baseline characteristics for each group of patients and anticoagulant of interest were described as percentages, means (SD), or medians (interquartile ranges). Incidence rates for each outcome were calculated based on the numbers with the outcome and the person years of follow-up, and were age and sex standardised for each drug. To estimate the risks associated with each DOAC, an outcome specific Cox model containing all confounding factors was used, with warfarin as a primary reference. To quantify differences between apixaban and other DOACs an additional analysis was run with apixaban as a reference.

To account for a log-normal distribution, logarithm of body mass index was used. Age was included using fractional polynomials. Patients with missing ethnicity data were included in the white category. To assess the validity of this assumption, a sensitivity analysis was run for ethnicity where the missing values were included as a separate category. Missing values for body mass index, smoking status, alcohol consumption, and systolic blood pressure were assumed as missing at random and imputed using chained equations.^36^ We used an outcome specific imputation model including outcome, length of follow-up, all confounders, anticoagulant type, and prescribed dose. Where possible, depending on numbers, we pooled the results obtained from QResearch and CPRD using a fixed effect model with inverse variance weights. Where any heterogeneity was detected, the results were combined using a random effect model.^37^ Because the CPRD sample was relatively small, not every outcome in the more disaggregated analyses yielded a sufficient number of events. This mainly occurred for the subcohort of patients without atrial fibrillation and in the dosage analyses. In these cases, results from QResearch alone were reported.

We carried out analyses for the cohort of all patients who started anticoagulants in the study period, with additional separate analyses for a subcohort with atrial fibrillation and the remaining subcohort with other indications for anticoagulant prescription. The main results presented are those for the two subcohorts separately, with the findings for the pooled cohort presented as supplementary material.

To estimate the absolute magnitude of risks associated with different DOACs when compared with warfarin, we calculated numbers needed to treat or harm using the adjusted hazard ratios and baseline rates for warfarin.^38^ Baseline rates were estimated by weighting rates from QResearch and CPRD. We calculated the numbers for 6, 12, 18, and 24 months after treatment commenced.

In addition to the sensitivity analysis for ethnicity described previously, three further sensitivity analyses were run. Being admitted to hospital for bleeding, ischaemic stroke, or venous thromboembolism may result in a switch of anticoagulant used without any subsequent GP records of the change. So patients who were admitted to hospital for one of these outcomes were censored at the time of the hospital stay in the analysis of other outcomes in a second sensitivity analysis. To assess the validity of the assumption that missing data were missing at random, a third sensitivity analysis was run only on patients with complete data.

The fourth sensitivity analysis, using propensity score weighting,^39^ was run on the subcohort with complete data. This approach has been used previously for studying DOACs in comparison with warfarin.^40^ Three separate propensity scores were developed. The first to predict the use of dabigatran among dabigatran and warfarin users. The second to predict use of rivaroxaban among rivaroxaban and warfarin users. The third to predict use of apixaban among apixaban and warfarin users. All available variables described as confounding factors were included in the development of the propensity scores. Patients with propensity scores from non-overlapping regions were excluded from the relevant analysis. Three separate Cox models were then run, where the use of each DOAC in turn was adjusted for the relevant propensity score.

### Patient involvement

Patient representatives from the QResearch Advisory Board wrote the information for patients on the QResearch website about the use of the database for research. Patients were not involved in setting the research question, the outcome measures, the design, or implementation of this study. Lay people and patient representatives were involved in writing and approving the lay summaries during the bid process. The patient representative in the publication review process expressed appreciation of the real world nature of the study, highlighting the usefulness of such studies for informing doctor-patient discussions.

## Results

### Cohort characteristics

**Fig 1:**
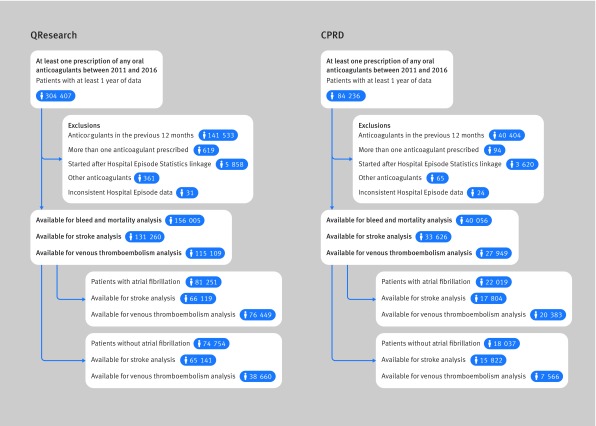
Flow of the included patients for QResearch and Clinical Practice Research Datalink (CPRD) analysis

Figure 1 shows that 156 005 patients from QResearch and 40 056 from Clinical Practice Research Datalink (CPRD), who started or restarted anticoagulants (after more than a 12 month gap) between 2011 and 2016, were eligible for inclusion. Overall, 53% were diagnosed with atrial fibrillation (81 251 in QResearch and 22 019 in CPRD) leaving 47% of patients prescribed anticoagulants for other indications (74 754 in QResearch and 18 037 in CPRD).

**Fig 2:**
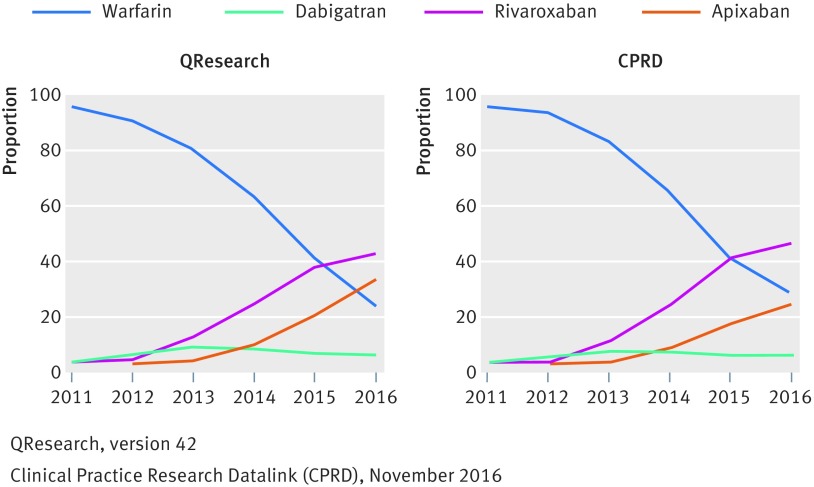
Proportion of patients prescribed different anticoagulants in each year by database

In the subcohort with atrial fibrillation, across the databases, there were 70 585 (68%) patients taking warfarin, 5537 (5%) taking dabigatran, 16 547 (16%) taking rivaroxaban, and 10 601 (10%) taking apixaban. In the subcohort without atrial fibrillation, there were 61 646 (66%) taking warfarin, 2207 (2%) taking dabigatran, 21 316 (23%) taking rivaroxaban, and 7622 (8%) taking apixaban. Figure 2 and supplementary table 2 show that although overall 67% of patients were prescribed warfarin, its use declined during the study period over both databases from 98% in 2011 to 23% in 2016. DOAC use had risen, from 1% to 42% for rivaroxaban and from 0% to 31% for apixaban. Dabigatran reached a peak in 2013 (10%) but dropped to 3% in 2016. This pattern was similar for patients with atrial fibrillation and without atrial fibrillation.

Table 1 and supplementary table 3 show the characteristics of patients with atrial fibrillation by database. Table 2 and supplementary table 4 show the characteristics of patients without atrial fibrillation by database. The tables show the consistency between the cohorts from the two databases apart from the slightly shorter exposure period in CPRD because of the shorter study inclusion period. Patients were exposed to warfarin for longer than to direct oral anticoagulants (DOACs) in both subcohorts, with a median exposure of 11 months in QResearch (9 months in CPRD) for the atrial fibrillation subcohort and six months (in both databases) for the subcohort without atrial fibrillation. In comparison, the DOACs had median duration of nine months in QResearch (five months in CPRD) for the atrial fibrillation subcohort, and three months in both QResearch and CPRD for the subcohort without atrial fibrillation.

**Table 1:** Patients with atrial fibrillation: selected baseline characteristics of patients and comorbidities in the QResearch and Clinical Practice Research Datalink (CPRD) cohorts. Values are percentages (numbers) unless stated otherwise

Characteristic	QResearch					CPRD			
	Warfarin	Dabigatran	Rivaroxaban	Apixaban		Warfarin	Dabigatran	Rivaroxaban	Apixaban
Total no of patients	53 921	4534	13 597	9199		16 664	1003	2950	1402
Median (interquartile range) days of treatment	344 (150-714)	271 (89-627)	265 (97-496)	248 (100-440)		286 (135-589)	214 (87-479)	163 (69-328)	143 (60-295)
Sex:									
Men	55.5 (29 913)	58.0 (2629)	54.4 (7391)	51.8 (4764)		55.7 (9278)	61.5 (617)	54.1 (1596)	54.9 (769)
Women	44.5 (24 008)	42.0 (1905)	45.6 (6206)	48.2 (4435)		44.3 (7386)	38.5 (386)	45.9 (1354)	45.1 (633)
Mean (SD) age at baseline	74.8 (10.4)	74.7 (10.7)	75.8 (10.9)	76.5 (10.9)		74.8 (10.3)	74.4 (10.8)	75.9 (10.8)	76.6 (10.9)
Comorbidities at baseline:									
Alcohol dependence	2.4 (1285)	3.2 (143)	2.9 (388)	3.1 (286)		2.2 (365)	2.9 (29)	2.8 (83)	3.4 (48)
Bleeding disorders	0.9 (486)	0.8 (38)	1.0 (136)	1.1 (102)		1.2 (193)	1.0 (10)	1.5 (43)	1.4 (20)
Cancer (any)	12.1 (6530)	12.5 (567)	13.3 (1806)	13.1 (1205)		12.4 (2073)	11.2 (112)	12.9 (382)	12.8 (179)
Chronic liver disease or pancreatitis	1.1 (582)	1.4 (62)	1.4 (187)	1.3 (120)		0.9 (155)	1.7 (17)	1.6 (46)	0.9 (13)
Chronic obstructive pulmonary disease	9.9 (5355)	8.4 (382)	9.7 (1314)	10.0 (920)		9.5 (1586)	9.6 (96)	9.7 (287)	8.1 (114)
Chronic renal disease	2.8 (1487)	1.0 (45)	1.6 (224)	2.1 (195)		2.9 (483)	1.5 (15)	2.0 (60)	1.9 (27)
Congestive cardiac failure	14.1 (7595)	11.1 (502)	11.4 (1553)	12.8 (1173)		13.4 (2227)	11.1 (111)	11.0 (324)	14.9 (209)
Coronary heart disease	25.3 (13 625)	22.0 (997)	22.1 (3005)	24.3 (2234)		25.5 (4251)	23.0 (231)	21.1 (622)	25.1 (352)
Diabetes	19.0 (10 255)	17.3 (784)	17.9 (2435)	19.3 (1772)		17.9 (2979)	16.7 (167)	18.0 (530)	19.0 (267)
Dyspepsia	18.0 (9684)	18.0 (817)	18.6 (2523)	19.1 (1759)		26.1 (4355)	25.4 (255)	26.9 (795)	24.8 (348)
Falls or hip fracture*	7.6 (4124)	7.1 (322)	7.5 (1015)	8.2 (756)		6.0 (992)	4.9 (49)	6.7 (199)	6.6 (92)
Hip or knee operation*	0.6 (319)	0.9 (40)	1.0 (131)	0.6 (59)		1.5 (246)	1.2 (12)	1.7 (51)	1.8 (25)
Hypertension	62.2 (33 555)	60.6 (2746)	59.2 (8053)	59.9 (5513)		62.3 (10 383)	59.9 (601)	62.1 (1833)	62.8 (881)
Ischaemic stroke†	18.1 (9752)	22.0 (999)	16.8 (2290)	22.7 (2091)		18.0 (3001)	23.5 (236)	19.7 (582)	28.2 (396)
Oesophageal varices	0.1 (51)	0.1 (6)	0.1 (11)	0.1 (11)		0.1 (9)	NA	NA	NA
Peptic ulcer	7.5 (4065)	7.4 (336)	7.1 (971)	8.4 (772)		8.3 (1380)	7.9 (79)	8.8 (260)	9.6 (135)
Valvular heart disease	12.2 (6553)	9.4 (428)	8.8 (1191)	10.6 (975)		9.8 (1630)	7.2 (72)	6.6 (196)	9.1 (127)
Venous thromboembolism†	6.4 (3450)	3.0 (138)	6.1 (830)	4.2 (384)		7.7 (1280)	5.6 (56)	7.3 (216)	6.0 (84)
Previous bleed:									
Any†	23.8 (12 848)	25.9 (1176)	26.0 (3541)	27.1 (2493)		28.0 (4674)	29.5 (296)	29.5 (869)	29.7 (417)
Intracranial†	0.8 (435)	1.2 (54)	1.1 (146)	1.6 (143)		1.1 (191)	1.9 (19)	1.4 (41)	2.1 (30)
Haematuria	10.9 (5883)	12.9 (583)	12.2 (1665)	12.1 (1110)		12.1 (2018)	12.0 (120)	12.0 (354)	11.4 (160)
Haemoptysis†	2.6 (1428)	2.3 (106)	2.7 (364)	2.7 (247)		3.6 (599)	3.4 (34)	3.3 (97)	3.2 (45)
Previous gastrointestinal bleed:†									
All	12.6 (6785)	13.6 (615)	13.8 (1878)	14.8 (1360)		15.7 (2610)	16.7 (168)	17.1 (504)	18.3 (256)
Upper	4.2 (2260)	4.5 (203)	4.7 (640)	5.1 (471)		5.1 (844)	5.1 (51)	6.2 (184)	6.0 (84)
Lower	9.4 (5067)	10.4 (472)	10.3 (1394)	10.9 (1004)		12.0 (2004)	12.8 (128)	12.9 (381)	14.0 (196)
Other drugs:									
Proton pump inhibitors	43.4 (23 375)	44.1 (2000)	41.1 (5593)	44.1 (4053)		41.4 (6894)	41.3 (414)	42.1 (1243)	42.4 (594)
Antibiotics‡	10.1 (5460)	8.7 (396)	7.5 (1015)	6.8 (622)		9.0 (1495)	6.9 (69)	7.8 (231)	5.1 (71)
Antiplatelet	29.9 (16 135)	23.3 (1055)	19.8 (2694)	17.4 (1602)		40.2 (6705)	39.1 (392)	31.0 (914)	33.5 (469)
Antidepressants	15.7 (8444)	14.8 (669)	15.4 (2095)	16.8 (1550)		14.5 (2420)	12.9 (129)	17.3 (510)	17.1 (240)
Anticonvulsants	0.8 (413)	0.4 (20)	0.6 (85)	0.8 (69)		0.8 (126)	0.5 (5)	0.9 (26)	0.9 (12)
NSAIDs	6.9 (3709)	8.5 (386)	7.3 (994)	5.7 (523)		6.6 (1105)	8.3 (83)	6.6 (195)	6.1 (85)
Corticosteroids	12.3 (6633)	11.2 (506)	10.7 (1450)	9.9 (914)		10.9 (1824)	8.9 (89)	10.2 (300)	8.6 (120)
Statins	55.2 (29 763)	53.6 (2428)	51.3 (6972)	54.1 (4975)		53.4 (8904)	52.9 (531)	51.2 (1509)	56.0 (785)
Hormones (women)	1.5 (370)	2.3 (43)	1.5 (93)	1.3 (57)		2.7 (199)	2.1 (8)	3.0 (40)	2.8 (18)

For information on age distribution, ethnicity, smoking, alcohol consumption, and Townsend quintiles see supplementary table 3.

NA=not applicable, fewer than 5 observations; NSAIDs=non-steroidal anti-inflammatory drugs.

* Within the last 6 months.

† Based on general practice and Hospital Episode Statistics records.

‡ Within the last 6 months before the drug start date.

**Table 2:** Patients without atrial fibrillation: selected baseline characteristics of patients and comorbidities in the QResearch and Clinical Practice Research Datalink (CPRD) cohorts. Values are percentages (numbers) unless stated otherwise

Characteristic	QResearch					CPRD			
	Warfarin	Dabigatran	Rivaroxaban	Apixaban		Warfarin	Dabigatran	Rivaroxaban	Apixaban
Total no of patients	47 331	1868	18 423	7132		14 315	339	2893	490
Median (interquartile range) days of treatment	196 (111-138)	88 (58-244)	89 (51-196)	115 (58-215)		193 (114-308)	78 (57-240)	86 (45-183)	102 (51-228)
Sex:									
Men	53.6 (25 377)	53.1 (992)	48.8 (8985)	51.7 (3689)		53.2 (7615)	53.1 (180)	47.3 (1368)	50.2 (246)
Women	46.4 (21 954)	46.9 (876)	51.2 (9438)	48.3 (3443)		46.8 (6700)	46.9 (159)	52.7 (1525)	49.8 (244)
Mean (SD) age at baseline	66.5 (15.6)	71.6 (12.9)	68.2 (15.7)	73.9 (13.6)		66.3 (15.9)	71.6 (12.7)	66.9 (16.4)	74.7 (13.5)
Comorbidities at baseline:									
Alcohol dependence	3.1 (1469)	2.6 (49)	3.3 (599)	3.3 (236)		2.3 (333)	2.1 (7)	3.2 (94)	3.9 (19)
Bleeding disorders	1.4 (641)	0.7 (14)	1.2 (221)	1.0 (73)		1.6 (231)	NA	1.9 (54)	1.0 (5)
Cancer (any)	13.5 (6405)	10.8 (202)	13.1 (2408)	13.3 (949)		13.8 (1971)	12.1 (41)	13.7 (396)	13.9 (68)
Chronic liver disease or pancreatitis	1.5 (699)	1.1 (20)	1.2 (229)	1.4 (97)		1.6 (228)	NA	1.5 (43)	3.1 (15)
Chronic obstructive pulmonary disease	8.2 (3899)	7.8 (146)	7.9 (1457)	10.1 (719)		8.4 (1205)	4.1 (14)	8.4 (242)	8.8 (43)
Chronic renal disease	3.1 (1466)	1.0 (18)	1.3 (246)	2.2 (158)		3.2 (453)	NA	1.9 (55)	2.2 (11)
Congestive cardiac failure	7.5 (3540)	6.6 (124)	5.0 (919)	8.8 (625)		6.7 (964)	5.9 (20)	5.6 (162)	7.1 (35)
Coronary heart disease	17.9 (8471)	18.9 (353)	13.5 (2488)	22.5 (1605)		17.9 (2560)	18.6 (63)	13.9 (401)	26.3 (129)
Diabetes	15.1 (7143)	17.0 (318)	15.1 (2780)	20.0 (1425)		14.0 (2007)	13.6 (46)	14.0 (404)	19.4 (95)
Dyspepsia	17.3 (8166)	16.9 (316)	17.3 (3193)	17.8 (1272)		24.5 (3502)	21.8 (74)	25.2 (730)	26.3 (129)
Falls or hip fracture*	7.2 (3405)	17.0 (317)	8.3 (1538)	6.6 (472)		5.8 (824)	6.5 (22)	5.8 (167)	6.5 (32)
Hip or knee operation*	3.1 (1445)	23.0 (430)	7.2 (1318)	3.7 (261)		5.2 (738)	30.7 (104)	8.2 (237)	6.7 (33)
Hypertension	40.5 (19 184)	51.3 (959)	40.0 (7363)	52.1 (3714)		43.3 (6200)	48.7 (165)	42.4 (1226)	54.9 (269)
Ischaemic stroke†	12.0 (5661)	20.6 (384)	10.5 (1937)	22.9 (1631)		11.6 (1663)	23.0 (78)	10.7 (309)	33.7 (165)
Oesophageal varices	0.3 (137)	NA	0.1 (18)	0.1 (6)		0.2 (29)	NA	NA	NA
Peptic ulcer	6.5 (3097)	7.0 (131)	5.6 (1040)	7.2 (513)		6.8 (979)	5.6 (19)	6.7 (195)	9.4 (46)
Valvular heart disease	8.7 (4133)	5.8 (108)	4.3 (791)	7.1 (506)		6.9 (982)	6.5 (22)	3.6 (103)	6.5 (32)
Venous thromboembolism†	58.0 (27 464)	8.5 (159)	39.2 (7222)	17.5 (1249)		62.9 (9003)	10.3 (35)	45.9 (1329)	21.2 (104)
Previous bleed:									
Any†	22.3 (10 552)	22.7 (424)	23.3 (4301)	23.8 (1697)		25.7 (3672)	25.4 (86)	26.4 (763)	31.6 (155)
Intracranial†	1.1 (534)	1.1 (21)	1.1 (207)	1.2 (87)		1.2 (177)	1.8 (6)	1.6 (46)	2.2 (11)
Haematuria	9.2 (4377)	9.7 (181)	9.6 (1775)	10.1 (721)		10.0 (1431)	10.3 (35)	9.0 (261)	11.8 (58)
Haemoptysis†	2.8 (1305)	2.4 (44)	2.6 (486)	2.5 (179)		3.3 (474)	3.5 (12)	4.0 (115)	4.7 (23)
Previous gastrointestinal bleed:†									
All	12.1 (5716)	12.5 (233)	13.0 (2400)	13.4 (955)		14.8 (2124)	13.6 (46)	15.5 (449)	16.9 (83)
Upper	4.2 (1995)	4.0 (74)	4.1 (754)	4.9 (350)		4.8 (687)	6.5 (22)	5.6 (163)	7.6 (37)
Lower	8.8 (4186)	9.5 (177)	10.1 (1856)	9.7 (694)		11.4 (1639)	8.6 (29)	11.8 (342)	11.8 (58)
Other drugs:									
Proton pump inhibitors	42.8 (20 259)	45.0 (841)	40.4 (7434)	42.7 (3042)		40.4 (5777)	44.8 (152)	40.8 (1181)	46.5 (228)
Antibiotics^‡^	11.0 (5222)	8.7 (162)	8.6 (1576)	5.6 (397)		10.1 (1452)	8.3 (28)	9.6 (277)	7.1 (35)
Antiplatelet	20.7 (9797)	21.6 (403)	16.4 (3014)	17.8 (1273)		22.5 (3216)	27.7 (94)	17.6 (508)	28.8 (141)
Antidepressants	21.9 (10 352)	19.8 (370)	22.2 (4094)	19.8 (1411)		20.9 (2992)	19.8 (67)	23.0 (664)	22.9 (112)
Anticonvulsants	1.5 (696)	0.8 (15)	1.2 (217)	1.0 (72)		1.4 (195)	1.5 (5)	1.3 (39)	NA
NSAIDs	12.1 (5722)	17.2 (321)	13.2 (2434)	5.7 (406)		12.0 (1723)	23.6 (80)	12.5 (361)	7.8 (38)
Corticosteroids	13.5 (6407)	9.2 (172)	10.5 (1934)	9.4 (670)		12.7 (1822)	10.3 (35)	11.4 (330)	11.2 (55)
Statins	39.6 (18 726)	49.1 (917)	35.3 (6507)	51.2 (3655)		37.3 (5333)	49.9 (169)	33.9 (980)	52.7 (258)
Hormones (women)	2.5 (546)	2.3 (20)	2.7 (257)	1.7 (58)		6.7 (449)	5.7 (9)	8.5 (130)	3.3 (8)

For information on age distribution, ethnicity, smoking, alcohol consumption, and Townsend quintiles see supplementary table 4.

NA=not applicable, fewer than 5 observations; NSAIDs=non-steroidal anti-inflammatory drugs.

* Within the last 6 months.

† Based on general practice and Hospital Episode Statistics records.

‡ Within the last 6 months before the drug start date.

Table 1 and table 2 show that across both databases, patients with atrial fibrillation were older than patients without atrial fibrillation (mean age 75 *v* 66), had more age related comorbidities, and used more related drugs. More patients in the subcohort with atrial fibrillation than in the subcohort without atrial fibrillation had heart related diseases such as congestive cardiac failure (13% *v* 7%), coronary heart disease (25% *v* 17%), treated hypertension (62% *v* 42%), diabetes (19% *v* 15%), and previous ischaemic stroke (19% *v* 13%); a much lower proportion had venous thromboembolism (6% *v* 50%). The proportion of patients diagnosed with cancer, was slightly higher in the subcohort without atrial fibrillation (13.4%) than in the subcohort with atrial fibrillation (12.4%) in both databases.

In the subcohort with atrial fibrillation, patients prescribed different anticoagulants were of similar age (with means ranging between 74.4 and 76.6), but in the subcohort without atrial fibrillation, patients on warfarin were the youngest (overall mean 66.5) and patients on apixaban were the oldest (overall mean 74.0). Across both databases and both subcohorts, the proportion of patients with chronic renal disease was highest in the warfarin group (2.9%) and among the patients using DOACs was highest in the apixaban groups (on average 2.2%). Proportions of patients in the different ethnic, smoking status, and alcohol consumption categories, and quintiles of Townsend deprivation scores were broadly similar across subcohorts, types of anticoagulants, and databases (see supplementary tables 3 and 4).

### Incidence rates

Table 3 and table 4 show follow-up time and the number of events for subcohorts with and without atrial fibrillation respectively. Supplementary table 5 shows the data for all patients. The rates of major bleeding in the warfarin groups varied between 25.1 and 35.2 per 1000 person years. In warfarin users, the rates for gastrointestinal bleeding were higher in CPRD in both subcohorts.

**Table 3:** Patients with atrial fibrillation: age-sex standardised incidence rates per 1000 person years (py) of outcomes by database

Drug	QResearch				CPRD		
	Person years	No of events	Age-sex standardised rate per 1000 py (95% CI)		Person years	No of events	Age-sex standardised rate per 1000 py (95% CI)
**Major bleeding **							
Warfarin	72 487	1813	25.1 (24.0 to 26.3)		18 795	515	27.5 (25.1 to 29.8)
Dabigatran	4988	107	21.8 (17.7 to 26.0)		886	17	19.1 (10.0 to 28.3)
Rivaroxaban	12 515	338	26.5 (23.7 to 29.4)		1844	66	36.3 (27.4 to 45.1)
Apixaban	7471	119	15.4 (12.6 to 18.3)		768	22	29.0 (16.6 to 41.5)
**Intracranial bleed**							
Warfarin	73 776	448	6.2 (5.6 to 6.7)		19 080	112	5.9 (4.8 to 7.0)
Dabigatran	5082	14	3.0 (1.4 to 4.6)		894	<5	1.0 (0.0 to 3.0)
Rivaroxaban	12 668	66	5.1 (3.9 to 6.3)		1865	15	8.2 (4.0 to 12.5)
Apixaban	7508	22	2.6 (1.4 to 3.7)		774	<5	5.0 (0.0 to 10.0)
**Haematuria**							
Warfarin	73 105	585	8.0 (7.3 to 8.6)		18 948	158	8.3 (7.0 to 9.6)
Dabigatran	5040	33	6.4 (4.2 to 8.6)		890	7	7.3 (1.8 to 12.8)
Rivaroxaban	12 610	100	7.9 (6.4 to 9.5)		1853	21	11.6 (6.6 to 16.6)
Apixaban	7498	33	4.4 (2.9 to 5.9)		771	7	8.7 (2.1 to 15.2)
**Haemoptysis**							
Warfarin	73 755	107	1.4 (1.2 to 1.7)		19 069	27	1.4 (0.9 to 1.9)
Dabigatran	5067	8	1.4 (0.4 to 2.5)		894	<5	1.3 (0.0 to 3.8)
Rivaroxaban	12 669	18	1.4 (0.8 to 2.1)		1866	<5	1.2 (0.0 to 2.8)
Apixaban	7511	<5	0.5 (0.0 to 1.1)		774	<5	1.2 (0.0 to 3.6)
**All gastrointestinal bleed**							
Warfarin	73 360	691	9.5 (8.8 to 10.2)		18 978	224	11.8 (10.3 to 13.4)
Dabigatran	5047	54	11.2 (8.2 to 14.2)		890	8	9.4 (2.9 to 15.9)
Rivaroxaban	12 603	158	12.1 (10.2 to 14.1)		1858	30	16.0 (10.2 to 21.9)
Apixaban	7489	62	8.2 (6.1 to 10.2)		771	10	14.1 (5.2 to 23.0)
**Upper gastrointestinal bleed**							
Warfarin	73 424	617	8.5 (7.8 to 9.1)		18 989	204	10.7 (9.3 to 12.2)
Dabigatran	5047	53	11.0 (8.0 to 14.0)		891	7	8.0 (2.1 to 14.0)
Rivaroxaban	12 612	149	11.5 (9.6 to 13.3)		1858	29	15.5 (9.8 to 21.2)
Apixaban	7491	58	7.6 (5.6 to 9.7)		772	9	12.6 (4.2 to 20.9)
**Rectal bleed**							
Warfarin	73 769	78	1.1 (0.8 to 1.3)		19 081	22	1.2 (0.7 to 1.7)
Dabigatran	5082	<5	0.3 (0.0 to 0.8)		893	<5	1.4 (0.0 to 4.0)
Rivaroxaban	12 670	9	0.7 (0.2 to 1.1)		1866	<5	0.6 (0.0 to 1.6)
Apixaban	7509	5	0.6 (0.1 to 1.2)		773	<5	1.5 (0.0 to 4.5)
**Ischaemic stroke**							
Warfarin	59 343	794	13.5 (12.6 to 14.5)		15 349	225	14.7 (12.8 to 16.6)
Dabigatran	3744	58	15.9 (11.8 to 20.1)		642	7	11.4 (2.7 to 20.2)
Rivaroxaban	10 278	128	12.0 (9.9 to 14.1)		1434	34	23.6 (15.5 to 31.7)
Apixaban	5573	86	15.2 (11.9 to 18.5)		535	9	16.4 (5.5 to 27.3)
**Venous thromboembolism**							
Warfarin	69 569	215	3.1 (2.7 to 3.5)		17 676	68	3.8 (2.9 to 4.8)
Dabigatran	4921	6	1.2 (0.2 to 2.2)		846	<5	1.3 (0.0 to 3.9)
Rivaroxaban	11 992	50	4.1 (2.9 to 5.2)		1726	12	6.7 (2.8 to 10.6)
Apixaban	7230	19	2.5 (1.3 to 3.6)		726	5	6.8 (0.6 to 13.0)
**Mortality**							
Warfarin	73 839	3183	44.6 (43.0 to 46.1)		19 094	780	41.7 (38.7 to 44.6)
Dabigatran	5083	212	43.1 (37.3 to 49.0)		894	38	41.9 (28.4 to 55.5)
Rivaroxaban	12 679	757	54.6 (50.6 to 58.6)		1866	112	53.2 (42.9 to 63.4)
Apixaban	7511	472	53.5 (48.4 to 58.5)		774	56	61.9 (45.0 to 78.9)

**Table 4:** Patients without atrial fibrillation: age-sex standardised incidence rates per 1000 person years (py) of outcomes by database

Drug	QResearch				CPRD		
	Person years	No of events	Age-sex standardised rate per 1000 py (95% CI)		Person years	No of events	Age-sex standardised rate per 1000 py (95% CI)
**Major bleeding**							
Warfarin	39 335	1132	29.2 (27.5 to 30.9)		10 796	378	35.2 (31.6 to 38.7)
Dabigatran	1129	33	31.0 (18.8 to 43.1)		183	6	28.6 (4.2 to 53.0)
Rivaroxaban	8066	238	29.4 (25.6 to 33.1)		1143	41	34.9 (24.0 to 45.7)
Apixaban	3273	71	18.3 (13.6 to 23.1)		219	<5	5.9 (0.0 to 13.0)
**Intracranial bleed**							
Warfarin	39 929	244	6.3 (5.5 to 7.1)		10 952	78	7.2 (5.6 to 8.8)
Dabigatran	1137	<5	2.9 (0.0 to 5.8)		184	<5	3.5 (0.0 to 10.3)
Rivaroxaban	8155	29	3.5 (2.2 to 4.8)		1156	<5	2.7 (0.0 to 5.4)
Apixaban	3297	19	5.2 (2.7 to 7.7)		220	0	NA
**Haematuria**							
Warfarin	39 685	351	8.9 (8.0 to 9.8)		10 897	109	10.0 (8.1 to 11.9)
Dabigatran	1133	8	7.9 (2.0 to 13.8)		184	<5	8.4 (0.0 to 25.0)
Rivaroxaban	8119	71	9.0 (6.9 to 11.1)		1150	11	9.2 (3.7 to 14.7)
Apixaban	3291	21	4.3 (2.4 to 6.1)		219	<5	3.0 (0.0 to 7.2)
**Haemoptysis**							
Warfarin	39 912	65	1.6 (1.2 to 2.0)		10 950	24	2.2 (1.3 to 3.0)
Dabigatran	1137	<5	2.3 (0.0 to 5.5)		184	0	NA
Rivaroxaban	8151	16	1.9 (1.0 to 2.9)		1155	<5	3.8 (0.1 to 7.6)
Apixaban	3300	<5	0.3 (0.0 to 0.8)		220	0	NA
**All gastrointestinal bleed**							
Warfarin	39 684	485	12.4 (11.3 to 13.5)		10 885	171	15.9 (13.5 to 18.2)
Dabigatran	1133	19	17.7 (8.1 to 27.4)		184	<5	16.6 (0.1 to 33.1)
Rivaroxaban	8114	126	15.2 (12.5 to 17.9)		1150	22	18.8 (10.8 to 26.9)
Apixaban	3286	31	8.8 (5.2 to 12.3)		220	<5	2.9 (0.0 to 8.6)
**Upper gastrointestinal bleed**							
Warfarin	39 719	431	11.1 (10.0 to 12.1)		10 896	152	14.1 (11.9 to 16.4)
Dabigatran	1134	16	15.6 (6.3 to 24.9)		184	<5	16.6 (0.1 to 33.1)
Rivaroxaban	8116	117	14.0 (11.5 to 16.6)		1150	22	18.8 (10.8 to 26.8)
Apixaban	3288	29	8.0 (4.6 to 11.4)		220	<5	2.9 (0.0 to 8.6)
**Rectal bleed**							
Warfarin	39 917	62	1.6 (1.2 to 2.0)		10 949	21	1.9 (1.1 to 2.7)
Dabigatran	1136	<5	2.1 (0.0 to 4.6)		184	0	NA
Rivaroxaban	8155	9	1.1 (0.4 to 1.9)		1156	0	NA
Apixaban	3298	<5	0.7 (0.0 to 1.8)		220	0	NA
**Ischaemic stroke**							
Warfarin	34 121	371	11.2 (10.1 to 12.4)		9459	109	11.6 (9.4 to 13.8)
Dabigatran	755	19	20.8 (10.7 to 30.9)		117	<5	21.1 (0.0 to 45.1)
Rivaroxaban	6996	83	11.8 (9.2 to 14.3)		990	9	7.9 (2.6 to 13.3)
Apixaban	2311	44	15.4 (10.6 to 20.3)		121	<5	17.5 (0.0 to 37.3)
**Venous thromboembolism **							
Warfarin	18 496	766	41.0 (38.1 to 44.0)		4526	182	40.0 (34.2 to 45.9)
Dabigatran	1055	10	9.7 (3.5 to 15.9)		166	6	35.1 (5.0 to 65.1)
Rivaroxaban	4001	688	180.3 (166.5 to 194.1)		532	112	239.7 (193.7 to 285.8)
Apixaban	2748	89	44.0 (33.4 to 54.7)		188	<5	11.7 (0.0 to 25.0)
**Mortality**							
Warfarin	39 960	2226	58.4 (56.0 to 60.8)		10 963	606	56.6 (52.1 to 61.1)
Dabigatran	1137	75	67.4 (41.7 to 93.0)		184	14	60.1 (26.9 to 93.2)
Rivaroxaban	8158	758	87.1 (80.8 to 93.3)		1156	130	108.4 (89.3 to 127.6)
Apixaban	3301	312	72.8 (63.9 to 81.7)		220	21	86.2 (25.4 to 146.9)

NA=not applicable.

In the subcohort without atrial fibrillation, the rates of different bleeds were generally slightly lower in QResearch than CPRD, although the number of events were too low for comparison. In the subcohort without atrial fibrillation in both QResearch and CPRD, the highest rates of venous thromboembolism were in patients taking rivaroxaban (180 and 240 per 1000 person years, respectively).

Table 3 and table 4 show that the mortality rates were consistently higher in patients without atrial fibrillation (from 58 to 87 per 1000 person years in QResearch and from 57 to 108 in CPRD) than in patients with atrial fibrillation (43 to 55 in QResearch and 42 to 62 in CPRD).

Overall, there was good consistency between the databases. For 120 combinations of subgroup, outcome, and drug, there were only eight combinations where rate pairs differed by more than 1 per 100 person years.

### Associations with anticoagulant exposure

**Fig 3:**
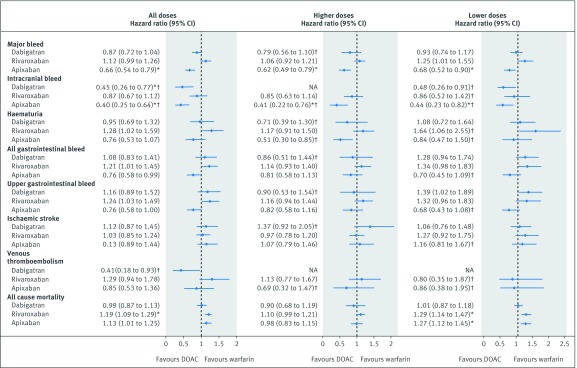
Patients with atrial fibrillation: adjusted Cox hazard ratios (95% confidence interval) for outcomes associated with exposure to study drugs overall and by prescribed dose compared with warfarin. NA=not available. *P value<0.01. †The results were only available from the QResearch database.

**Fig 4:**
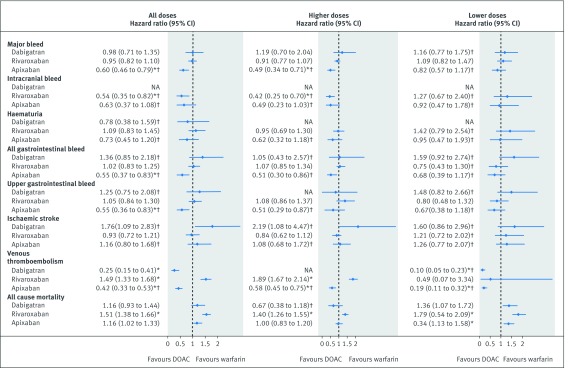
Patients without atrial fibrillation: adjusted Cox hazard ratios (95% confidence interval) for outcomes associated with exposure to study drugs overall and by prescribed dose compared with warfarin. NA=not available. *P value<0.01. †The results were only available from the QResearch database.

**Fig 5:**
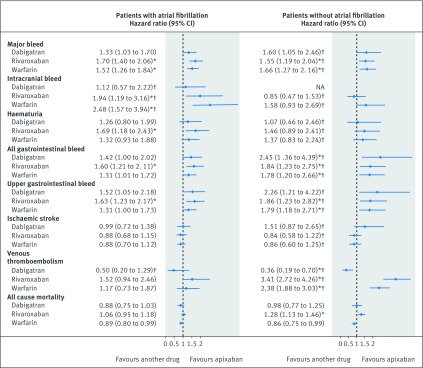
Patients with and without atrial fibrillation: adjusted Cox hazard ratios (95% confidence interval) for outcomes associated with exposure to study drugs compared with apixaban. NA=not available. *P value<0.01. †The results were only available from the QResearch database.

Figure 3 shows the results for patients with atrial fibrillation and figure 4 shows the results for patients without atrial fibrillation, with reference to warfarin. Figure 5 shows the results for both groups with reference to apixaban. Supplementary tables 5-7 show the adjusted hazard ratios in each of the two databases. Hazard ratios were adjusted for age, sex, ethnicity, smoking, alcohol, Townsend quintile, body mass index, systolic blood pressure, falls and hip fracture, hip or knee operations, comorbidities (alcoholism, atrial fibrillation, treated hypertension, chronic kidney disease, chronic obstructive pulmonary disease, liver disease, coronary heart disease, congestive cardiac failure, any cancer, or valvular peptic ulcer), previous events (bleed, venous thromboembolism, or ischaemic stroke), drugs at the baseline (macrolides, antiplatelets, anticonvulsant, corticosteroids, NSAIDs, statins, or hormones), and study year.

In patients with atrial fibrillation, apixaban was associated with a lower risk of major bleed than warfarin (adjusted hazard ratio 0.66, 95% confidence interval 0.54 to 0.79) (fig 3) and rivaroxaban (fig 5). Dabigatran (0.45, 0.26 to 0.77) and apixaban (0.40, 0.25 to 0.64) were associated with lower risks of intracranial bleed than warfarin, and rivaroxaban was associated with a higher risk compared to apixaban (1.94, 1.19 to 3.16). Although no drugs were significantly different from warfarin in risks of any other bleeding events, rivaroxaban was associated with higher risks compared with apixaban for haematuria, all gastrointestinal bleed and upper gastrointestinal bleed (fig 3 and fig 5).

In the subcohort without atrial fibrillation, apixaban was associated with lower risks of major bleed (adjusted hazard ratio 0.60, 95% confidence interval 0.46 to 0.79) than warfarin (fig 4) or rivaroxaban (fig 5). Rivaroxaban was associated with a lower risk of intracranial bleed (0.54, 0.35 to 0.82) compared with warfarin, and apixaban with lower risks of all gastrointestinal (0.55, 0.37 to 0.83) and upper gastrointestinal bleeds (0.55, 0.36 to 0.83). Dabigatran and rivaroxaban were associated with higher risks for all gastrointestinal bleeds compared with apixaban, rivaroxaban was also associated with a higher risk for upper gastrointestinal bleed (fig 5).

The risk of primary ischaemic stroke did not differ between any of the anticoagulants studied in either subcohort. Figure 4 shows that the risk of primary venous thromboembolism in patients with atrial fibrillation was not different between any drugs, but in the subcohort without atrial fibrillation compared with warfarin there was a higher risk in patients taking rivaroxaban (adjusted hazard ratio 1.49, 95% confidence interval 1.33 to 1.68) and lower risks in patients taking dabigatran (0.25, 0.15 to 0.41) and apixaban (0.42, 0.33 to 0.53).

For both patients with atrial fibrillation (adjusted hazard ratio 1.19, 95% confidence interval 1.09 to 1.29) and without atrial fibrillation (1.51, 1.38 to 1.66), the risk of mortality was increased in patients taking rivaroxaban compared with warfarin. Although the estimates for apixaban in both subcohorts were higher than unity (1.13, 1.01 to 1.26 for patients with atrial fibrillation; 1.16, 1.02 to 1.33 for patients without atrial fibrillation) and neither of them were statistically significant (at P<0.01), the estimate for the whole cohort was (adjusted hazard ratio 1.14, 95% confidence interval 1.05 to 1.24, P=0.001) (see supplementary figure 1 and supplementary table 5). Supplementary table 8 shows that most of these deaths were owing to causes other than bleeding, ischaemic stroke, or venous thromboembolism (91% in QResearch and 88% in CPRD).

### Numbers needed to harm and treat

Table 5 shows the number needed to treat or number needed to harm to measure the relative benefits or risks of DOACs in comparison with warfarin. In the subcohort with atrial fibrillation, over six months, the lowest number needed to treat (to avoid one extra major bleed) was for apixaban (182, 95% confidence interval 137 to 299). The lowest number needed to harm (to observe one extra death) over six months was for rivaroxaban (202, 131 to 410). In the subcohort without atrial fibrillation, over six months, the lowest number needed to treat to avoid one extra major bleed was also for apixaban (138, 102 to 207). The lowest number needed to harm for deaths was again for rivaroxaban (61, 47 to 82).

**Table 5:** Number needed to treat or harm (95% confidence interval) compared with warfarin

Outcome	6 months	12 months	18 months	24 months
**With atrial fibrillation**				
Numbers needed to treat:				
Major bleeding, apixaban	182 (137 to 299)	104 (78 to 170)	76 (58 to 126)	60 (45 to 99)
Intracranial bleed, dabigatran	545 (407 to 1310)	274 (204 to 658)	196 (146 to 472)	150 (111 to 360)
Intracranial bleed, apixaban	501 (401 to 828)	252 (201 to 416)	180 (144 to 298)	137 (110 to 227)
Numbers needed to harm:				
Mortality, rivaroxaban	202 (131 to 410)	118 (76 to 239)	86 (56 to 175)	70 (45 to 141)
**Without atrial fibrillation**				
Numbers needed to treat:				
Major bleeding, apixaban	138 (102 to 257)	85 (62 to 158)	61 (45 to 114)	49 (36 to 91)
Intracranial bleed, rivaroxaban	592 (423 to 1528)	323 (230 to 834)	224 (160 to 579)	185 (132 to 479)
All gastrointestinal bleed, apixaban	293 (207 to 756)	181 (128 to 467)	126 (89 to 326)	96 (68 to 248)
Upper gastrointestinal bleed, apixaban	329 (232 to 891)	200 (141 to 543)	138 (97 to 375)	108 (76 to 294)
Venous thromboembolism,* dabigatran	34 (30 to 43)	32 (28 to 40)	30 (27 to 39)	29 (26 to 37)
Venous thromboembolism,* apixaban	44 (38 to 55)	41 (35 to 51)	40 (34 to 49)	38 (33 to 47)
Numbers needed to harm:				
Venous thromboembolism,* rivaroxaban	53 (38 to 80)	49 (36 to 75)	48 (34 to 72)	46 (33 to 69)
Mortality, rivaroxaban	61 (47 to 82)	37 (29 to 49)	27 (21 to 37)	23 (18 to 30)

The calculations are based on the hazard ratios derived from QResearch or combined analysis. Only statistically significant associations between the exposure and outcome are included.

* Based on patients without venous thromboembolism before the start of anticoagulant

### Dose analysis

Overall, patients on lower doses of DOACs were older, had more comorbidities, previous events, and other drugs than patients on higher doses (see supplementary tables 9 and 10). In the subcohort with atrial fibrillation, patients on lower doses were on average 10 years older (mean 83 years *v* 73 across the databases), more likely to be women (58% *v* 41%), more likely to be non-drinkers (42% *v* 30%), more likely to have lower body mass index (mean 27 kg/m^2^ *v* 29 kg/m^2^), and more likely to have age associated morbidities, including hypertension (67% *v* 57%), congestive cardiac failure (17% *v* 10%), coronary heart disease (29% *v* 20%), valvular heart disease (12% *v* 8%), and chronic renal disease (4% *v* 1%). Patients on lower doses were also more likely to have had falls or hip fracture (12% *v* 6%) (see supplementary tables 9 and 10).

In the subcohort without atrial fibrillation, patients on lower doses of DOACs were on average 7 years older (mean 75 years *v* 68 across the databases), more likely to be women (59% *v* 48%), more likely to be non-drinkers (40% *v* 34%), and more likely to have age associated comorbidities, with diagnoses of hypertension (52% *v* 41%), congestive cardiac failure (9% *v* 5%), coronary heart disease (20% *v* 15%), valvular heart disease (7% *v* 4%), and chronic renal disease (3% *v* 1%) than patients on higher doses. Patients on lower doses were also more likely to have had falls or hip fracture (16% *v* 5%), hip or knee replacement operations (22% *v* 3%), and previous ischaemic stroke (18% *v* 13%). The proportion of patients with previous venous thromboembolism was lower than in the higher dose group (15% *v* 38%) (see supplementary tables 9 and 10).

Age-sex standardised rates for patients on lower and higher doses and adjusted hazard ratios with reference to warfarin are shown for the subcohort with atrial fibrillation in supplementary table 11 and for the subcohort without atrial fibrillation in supplementary table 12. Although higher doses were mainly associated with lower risks than lower doses, the confidence intervals for the adjusted hazard ratios overlapped for most outcomes and drugs (see fig 3 for patients with atrial fibrillation, fig 4 for patients without atrial fibrillation, and supplementary table 13 with supplementary figure 1 for all patients). In patients with atrial fibrillation, only low doses of rivaroxaban (adjusted hazard ratios of 1.29, 95% confidence interval 1.14 to 1.47) and apixaban (1.27, 1.12 to 1.45) were associated with an increased risk of mortality. In patients without atrial fibrillation, however, both low and high doses of rivaroxaban were associated with increased risks while for apixaban only low doses were associated with increased risk of mortality (1.34, 1.13 to 1.58).

### Sensitivity analyses

Analyses for ethnicity, where unrecorded values were included as a separate category, also obtained very similar results. Reanalysis of the whole cohort, but with patients censored if admitted to hospital for bleeding, ischaemic stroke, or venous thromboembolism, gave results which were very similar to the main analysis for all outcomes (see supplementary table 14). Results from the complete case analysis were comparable to the main analysis (see supplementary table 15). Analyses adjusted with propensity scores also resulted in similar hazard ratios compared with the complete case analysis (see supplementary table 15).

## Discussion

Our study, based on routinely collected care data, showed a decreased risk of major bleeding events associated with the use of apixaban compared with warfarin in both patients with atrial fibrillation and without atrial fibrillation. Similarly, in patients with atrial fibrillation, a lower risk of intracranial bleed was associated with dabigatran and apixaban. In patients without atrial fibrillation, use of rivaroxaban was associated with a lower risk of intracranial bleed and apixaban was associated with lower risks of any gastrointestinal bleed and upper gastrointestinal bleeds. In both patients with atrial fibrillation and without atrial fibrillation, rivaroxaban and lower doses of apixaban were associated with an increased risk of all cause mortality compared with warfarin.

### Strengths and weaknesses of this study

The study, using the two largest primary care databases in the UK to deliver high statistical power, contributes to the evidence from other major studies. The general practice records were linked to hospital and mortality data, so all recorded outcomes were identified. Consistency in records of comorbidities, lifestyle, and prescribing across the databases also facilitated the combination of results from each, so delivering narrower confidence intervals for our estimations.

An important limitation for our study and all earlier observational studies is the lack of information on patient adherence to their prescribed drugs, which may lead to possible misclassifications of exposure. It is not known when exactly a patient stopped taking anticoagulants, and our setting of 30 days as a period during which they could still have been exposed was selected primarily to make our study consistent with – and therefore comparable to – previous research. Warfarin has been shown to have the highest non-persistence and apixaban and rivaroxaban the lowest.^41^ A study based on routinely collected data has shown that adding international normalisation ratio information, which we could not use directly because of inconsistent recording, could increase the estimate of exposure to vitamin K antagonists by 13% to 18% over 12 months.^42^ In our sample, the median duration of exposure to warfarin was less than a year, so our addition of 30 days to exposure will to some extent have compensated for this lack of information regarding warfarin exposure. However, despite the addition of this 30 day grace period to each anticoagulant course, there is still uncertainty about precise periods of exposure.

The effect of non-adherence on bleeding rates has also been shown using commercial insurance data and non-adherence is likely to have contributed to various extents to underestimation of the efficacy of any of the drugs in our study with respect to the prevention of ischaemic stroke or venous thromboembolism.^43^ With respect to mortality outcomes, a greater proportion of the older patients on apixaban and rivaroxaban may have died while still taking anticoagulants, but from age related causes other than ischaemic stroke or venous thromboembolism. We decided to adjust for a diagnosis of chronic kidney disease in the analysis rather than undertake a detailed analysis of renal function through the analysis of individual blood tests. A reduced dose of direct oral anticoagulant (DOAC) is recommended in patients with renal impairment as well as for patients aged over 80 and under 60 kg and there were more patients diagnosed with chronic renal disease in all lower dose DOAC groups, particularly in the apixaban and rivaroxaban groups. We adjusted for renal disease and for age and body mass index, but renal disease and use of anticoagulants may still contribute to mortality rate and this needs further research.^44^

An increased risk of bleeding in patients taking warfarin compared to those taking a DOAC could be because of the regular monitoring required for warfarin users. Bleeds could be more likely to be detected in these patients than in those taking DOACs, introducing a surveillance bias. Our definition of the outcome as any bleed requiring admission to hospital or causing death makes it less likely that these would be missed in the patients taking DOACs. However, a minor bleed detected in warfarin users could have been treated before it developed into a more serious one.

Included patients had different indications for anticoagulation and the DOAC groups were generally older and less healthy than the comparator warfarin group. Extensive adjustment for confounders, however, should have helped to reduce possible indication bias.

Exposure in our study was based only on GP records, without information from other possible sources of anticoagulants such as anticoagulant clinics or hospital stays. A small proportion of patients might have had private health insurance with prescriptions not available on the GP records. In the UK, however, the overwhelming proportion of events included as outcomes in this study would not be treated using private medical care. There is also some uncertainty surrounding venous thromboembolism diagnoses in QResearch and Clinical Practice Research Datalink (CPRD) cohorts because the results of diagnostic tests are not available to researchers in primary care records. This might lead to a misclassification of the outcome and a slightly increased rate of venous thromboembolism. It may, however, happen to patients taking any anticoagulant and we are not aware of any systematic differences between the prescribing of these drugs, but we accept a possible shift in results towards unity. The findings regarding risk of venous thromboembolism associated with different anticoagulants should, however, be interpreted with caution.

These uncertainties could have affected our results in several ways. We may have included some patients who had had exposure to anticoagulants in the 12 months before their entry. Included patients admitted to hospital for bleeding events might also have stopped anticoagulant therapy and then suffered an ischaemic stroke, developed venous thromboembolism, or died, so causing their misclassification as anticoagulant users. Our sensitivity analysis censoring such patients did not, however, require alterations to our conclusions. We also lacked information about over-the-counter purchases of other drugs such as a non-steroidal anti-inflammatory drug or aspirin, but this is likely to have affected only a small number of patients.

Between QResearch and CPRD most of the results were consistent, but there were a few differences in rates and hazard ratios. This is not unexpected, partly owing to small numbers for some comparisons and because contributing practices for the two databases not only use different computer systems for data collection but also have somewhat different profiles in terms of their location within different geographical regions.^45^

This is an observational study with high quality information on which drugs have been prescribed, the dates and duration. However, limitations include lack of information on adherence and all the indications for prescribing. Although many adjustments have been done using the data available on the existing databases, there is a possibility of unmeasured confounding or confounding by indication. Routinely collected data are also not always consistently recorded and information stored in free text format is not extracted from the GP systems. Only available, consistently recorded variables can be used in such studies, which always creates the possibility of some residual confounding.

### Strengths and weaknesses in relation to other studies

Incidence rates of outcomes in general for patients taking anticoagulants depend on a number of study design factors. One is inclusion criteria, with incidence rates being lower for cohorts excluding patients with previous events. Another, the duration of the grace period after a prescription ends but when the patient is still considered exposed, may result in incidence rates being lower in studies with a shorter grace period. Grace periods were not consistent across the studies, ranging from three to 30 days, with studies in Denmark assuming continuous treatment.^18^ ^22^ Our rates were much higher than the rates from the Danish studies and from studies using US insurance data.^12^ ^15^ ^16^ ^20^

This was a large comprehensive study using the most recent data, so one of the study strengths is its representativeness in terms of new users (or restarters) of anticoagulant drugs. All data were routinely collected and included not only comorbidities and any drugs but information on lifestyle factors such as smoking and alcohol – not commonly used in previous studies.^12^ ^15^ ^17^ ^18^ ^21^ ^22^

Atrial fibrillation is one of the most common indications for anticoagulant prescribing, so almost all observational studies provide evidence for this restricted group. Approximately the same numbers of patients without atrial fibrillation are, however, also prescribed anticoagulants, creating a gap in knowledge about the effects of these drugs. Such patients are different in their comorbidities and indications for prescribing, so the risks of ischaemic stroke, venous thromboembolism, and mortality are unlikely to be the same.

It is difficult to discern the precise indications for anticoagulation. Not every patient diagnosed with atrial fibrillation is prescribed anticoagulants.^46^ Some patients in the atrial fibrillation subcohort also had hip fractures or operations which could have required anticoagulation. We believe that our findings for all anticoagulant users, although presented separately for patients with and without atrial fibrillation, provide more generalisable evidence than findings based only on the subset of patients with atrial fibrillation. For patients without atrial fibrillation, however, presenting aggregated results can only highlight overall risks associated with DOAC drugs without being able to be more specific about underlying associations between different drugs and different conditions.

To facilitate comparison with other studies, our study offers analyses separately for patients with atrial fibrillation and without atrial fibrillation, and for patients on different DOAC doses. Although we used a proportional hazard model adjusting for all available confounding factors, we also undertook a sensitivity analysis using the propensity score method and obtained very similar results.

### Important similarities and differences in results

Although patients with valvular heart disease were excluded from some trials and observational studies for patients with atrial fibrillation, a meta-analysis has shown that DOAC risks compared with warfarin for bleeding, ischaemic stroke, or systemic embolism and for death were similar for patients with atrial fibrillation with or without valvular heart disease.^47^ For the main outcome of major bleeding, results from our study for the subcohort with atrial fibrillation were consistent with existing evidence from randomised controlled trials.^11^ Apixaban appeared to be associated with the lowest risk of major bleeding in most of the larger studies.^12^ ^14^ ^18^ ^20^ ^21^ The risk of mortality in our subcohort with atrial fibrillation was similar for warfarin, dabigatran, and apixaban but elevated for rivaroxaban. Like the Danish study,^22^ our risk of mortality in this subcohort was elevated only for patients on lower doses of apixaban and rivaroxaban. The other Danish study of standard dosage showed decreased mortality for apixaban,^18^ but our findings showed equivalent risk to warfarin for such patients.

The risk of ischaemic stroke associated with DOACs in our subcohort of patients with atrial fibrillation was equivalent to warfarin, which is in line with the latest meta-analysis for prevention of ischaemic stroke and both Danish studies.^11^ ^18^ ^22^ Similarly, we did not show any different risks of venous thromboembolism for any DOACs compared with warfarin in patients with atrial fibrillation, which is also in line with the relevant findings from the latest meta-analysis.^11^

### Meaning of the study: possible explanations and implications for clinicians and policy makers

Anticoagulants are prescribed for a wide range of indications although the adverse events have been studied mostly in patients with atrial fibrillation.^12^ ^13^ ^14^ ^15^ ^16^ ^17^ ^18^ ^19^ ^20^ ^21^ ^22^ ^23^ ^24^ Our study has shown that the risk of major bleeding is lower in patients taking apixaban regardless of the reason for prescribing. This was most pronounced for intracranial bleeding in patients with atrial fibrillation and for gastrointestinal bleeding in patients without atrial fibrillation, appearing, in general, to show apixaban to be the safest drug.

Increased risk of all cause mortality was found in rivaroxaban users for both patients with atrial fibrillation and without atrial fibrillation. Apixaban was associated with an increased risk of all cause mortality in patients with atrial fibrillation and without atrial fibrillation, but only in patients on lower doses. The increased all cause mortality may be reflecting the closer monitoring of patients undergoing treatment with warfarin may be related to unmeasured confounding due to prescribing choices related to underlying comorbidities.

### Unanswered questions and future research

The use of DOACs in patients with atrial fibrillation has been extensively studied but this group represents only half of anticoagulant users. Our study provides the evidence for this group and highlights increased all cause mortality in the group of patients without atrial fibrillation indications for anticoagulant prescribing. This group, however, includes patients undergoing preventative treatment for venous thromboembolism or ischaemic stroke after hip or knee replacements, fractures, or other operations and studying this group in detail would require further splitting.

We were unable to investigate the risks of ischaemic stroke and venous thromboembolism in patients who had already experienced a prior event because it can be difficult to distinguish new events from ongoing reviews of previous events in electronic health records. The risk of bleeding was lower in patients taking DOACs but the risk of mortality was increased in rivaroxaban and lower dose apixaban users. This also requires further investigation.

### Conclusion

This large observational study, based on a general population in a primary care setting, provides reassurance about the safety of DOACs as an alternative to warfarin across all new incident users. Apixaban was found to be associated with a decreased risk of major bleeding, particularly for intracranial and gastrointestinal bleeds. This was consistent for patients with atrial fibrillation and without atrial fibrillation. Rivaroxaban and low dose apixaban were, however, associated with an increased risk of all cause mortality when compared with warfarin. Our results give an initial, reassuring, indication of the risk patterns for all patients taking anticoagulants, with respect to those prescribed apixaban.

**What is already known on this topic**

Randomised controlled trials of anticoagulants have shown the non-inferiority of direct oral anticoagulants (DOACs) compared with warfarin
Observational studies of anticoagulants, investigating outcomes in more real world environments, have mostly studied patients with atrial fibrillation
Studies including patients without atrial fibrillation have either predated the increase in use of DOACs, or have had incomplete patient selection or other study design weaknesses

**What this study adds**

Apixaban is associated with a decreased risk of major bleeding events in patients with atrial fibrillation and without atrial fibrillation compared with warfarin
Rivaroxaban is associated with a decreased risk of intracranial bleeds in patients without atrial fibrillation compared with warfarin
Rivaroxaban and low dose apixaban are associated with an increased risk of all cause mortality in patients with atrial fibrillation and without atrial fibrillation compared to warfarin

## Web Extra

Extra material supplied by the author

## Notes

Contributors: JHC initiated the study, undertook the original literature review, prepared the grant application, designed the study, drafted the study protocol, organised the extraction of the QResearch data, advised on clinical aspects of the study, interpreted the results, and critically reviewed the paper. CC contributed to the development of the idea, the study design, and advised on the analysis and interpretation of the results. TH did preliminary analyses of the data. YV reviewed the literature, contributed to the grant application and the study design, organised the extraction of Clinical Practice Research Datalink (CPRD) data, did the analysis on both datasets, and wrote the draft of the manuscript. JHC, CC, and TH critically reviewed the paper. YV is the guarantor of the study. All authors have approved the submitted version.

Ethical approval: The protocol for QResearch has been published in eprints and was reviewed in accordance with the requirements for the East Midlands Derby Research Ethic Committee (ref 03/4/021).^48^ The protocol for CPRD has been approved by The Independent Scientific Advisory Committee for MHRA Database Research (N 16_284R).

Data sharing: To guarantee the confidentiality of anonymised patient data and health information only the authors have had access to the data during the study in accordance with the relevant licence agreements. Access to the QResearch data are according to the information on the QResearch website (www.qresearch.org). Clinical Practice Research Datalink (CPRD) linked data were provided under a licence that does not permit sharing.

Transparency: The lead author (YV) affirms that the manuscript is an honest, accurate, and transparent account of the study being reported; that no important aspects of the study have been omitted and that any discrepancies from the study as planned have been explained.
