# Supplementary material for: Risks and benefits of direct oral anticoagulants versus warfarin in a real world setting: cohort study in primary care
Source: BMJ. 2018 Jul 4;362:k2505. doi: 10.1136/bmj.k2505 (PMC6031213; doi:10.1136/bmj.k2505)
Supplement: Supplementary file 1 — Supplementary information: Supplementary tables 1-15 [file viny041857.ww1.pdf]

Re: Risk and benefits of direct oral anticoagulants versus warfarin in real world setting: cohort studies using two primary care databases

Supplementary data

Content

eTable 1 ICD-10 codes for the outcomes 2
eTable 2 Supplementary data for Figure2: Number of patients starting anticoagulants by study year 2
eTable 3 **Patients with atrial fibrillation**: Characteristics of patients in the QResearch and CPRD cohorts, percentage (numbers). 3
eTable 4 **Patients without atrial fibrillation**: Characteristics of patients in the QResearch and CPRD cohorts, percentage (numbers). 5
eTable 5 **All patients**: Rates per 1000py of outcomes by drug and adjusted hazard ratios with reference to warfarin in QResearch and CPRD 7
eTable 6 **Patients with and without atrial fibrillation**: Adjusted hazard ratios (95% CI) for outcomes associated with exposure to study drugs compared with warfarin, by database 9
eTable 7 **Patients with and without atrial fibrillation**: Adjusted hazard ratios (95% CI) for outcomes associated with exposure to study drugs compared with **apixaban**, by database 11
eTable 8 Cause of death by drug and database, proportion of deceased patients on the drug 13
eTable 9 **Patients with and without atrial fibrillation**: Cohort characteristics by database, percentage (number) of patients on lower and higher doses for DOAC's 14
eTable 10 **Patients with and without atrial fibrillation**: Comorbidities, previous events and other medications, percentage (number) of patients on lower and higher doses for DOAC's. 16
eTable 11 **Patients with atrial fibrillation**: Rates per 1000py of outcomes by drug and dose for DOAC's and adjusted hazard rations with reference to **warfarin** in QResearch and CPRD 18
eTable 12 **Patients without atrial fibrillation**: Rates per 1000py of outcomes by drug and dose for DOAC's and adjusted hazard rations with reference to **warfarin** in QResearch and CPRD 20
eTable 13 **All patients**: Rates per 1000py of outcomes by drug and dose for DOAC's and adjusted hazard ratios with reference to warfarin in QResearch and CPRD 22
eTable 14 **All patients**: Sensitivity analysis, censoring patients at the time of hospitalisation related to other outcome. Adjusted hazard ratios (95% CI) for outcomes associated with exposure to study drugs compared with warfarin. 25
eTable 15 **All patients**: Complete case analysis and propensity score adjustment by database. Adjusted hazard ratios (95% CI) for outcomes associated with exposure to study drugs compared with warfarin. 26

eTable 1 ICD-10 codes for the outcomes

outcome	ICD10-code
Intracranial bleed	I60, I600, I601, I602, I603, I604, I605, I606, I607, I608, I609, I61, I610, I611, I612, I613, I614, I615, I616, I618, I619, I62, I620, I621, I629, S065, S066
Haematuria	R31, R31X
Haemoptysis	R04, R041, R042, R048, R049
Upper GI bleed	I850, K226, K250, K251, K252, K254, K255, K256, K260, K261, K262, K264, K265, K266, K270, K271, K272, K274, K275, K276, K279, K280, K281, K282, K284, K285, K286, K290, K920, K921, K922
Lower GI bleed	K625
Ischaemic stroke	G45, G450, G451, G452, G453, G454, G458, G459, I63, I630, I631, I632, I633, I634, I635, I636, I638, I639, I64, I64X
Venous thromboembolism	I26, I260, I269, I81, I81X, I82, I820, I822, I823, I828, I829

eTable 2 Supplementary data for Figure2: Number of patients starting anticoagulants by study year

	**QResearch**				**CPRD**			
Year	Warfarin	Dabigatran	Rivaroxaban	Apixaban	Warfarin	Dabigatran	Rivaroxaban	Apixaban
2011	21807	167	190		7841	54	42	
2012	23608	1055	607	13	7843	246	46	<5
2013	20538	1606	2611	336	6940	412	719	78*
2014	16622	1446	6109	1802	5161	352	1733	482
2015	12573	1204	11342	5701	2836	237	2715	1046
2016	6104	924	11161	8479	358	41	588	286

*) This figure includes patients for 2012 and 2013

*eTable 3 **Patients with atrial fibrillation**: Characteristics of patients in the QResearch and CPRD cohorts, percentage (numbers).*

	QResearch				CPRD			
	**warfarin**	**dabigatran**	**rivaroxaban**	**apixaban**	**warfarin**	**dabigatran**	**rivaroxaban**	**apixaban**
	% (n)	% (n)	% (n)	% (n)	% (n)	% (n)	% (n)	% (n)
Total N of patients	53921	4534	13597	9199	16664	1003	2950	1402
Age at baseline								
20-49	2.3 (1233)	2.2 (98)	1.8 (245)	1.6 (150)	2.2 (362)	1.9 (19)	1.8 (54)	1.6 (22)
50-59	5.9 (3201)	6.0 (274)	5.9 (798)	5.7 (521)	5.9 (986)	8.1 (81)	5.8 (170)	5.8 (82)
60-69	19.5 (10505)	21.1 (956)	19.2 (2613)	17.5 (1612)	19.5 (3246)	20.4 (205)	19.4 (572)	17.2 (241)
70-79	36.3 (19594)	35.2 (1595)	32.7 (4450)	31.7 (2912)	36.1 (6016)	35.9 (360)	32.2 (949)	32.2 (451)
80-89	31.2 (16831)	29.2 (1326)	31.8 (4327)	33.2 (3053)	32.0 (5326)	27.5 (276)	31.9 (942)	32.7 (459)
90 and over	4.7 (2557)	6.3 (285)	8.6 (1164)	10.3 (951)	4.4 (728)	6.2 (62)	8.9 (263)	10.5 (147)
Ethnicity recorded	84.2 (45426)	81.7 (3704)	80.9 (11000)	83.2 (7658)	96.4 (16066)	93.6 (939)	94.9 (2799)	95.3 (1336)
White or not recorded (UK or European)	97.0 (52308)	98.5 (4464)	97.1 (13205)	97.5 (8972)	97.9 (16319)	98.5 (988)	98.4 (2904)	97.9 (1372)
Ethnic minorities					2.1 (345)	1.5 (15)	1.6 (46)	2.1 (30)
Indian	0.7 (363)	0.4 (17)	0.7 (101)	0.5 (48)				
Pakistani	0.3 (167)	0.2 (7)	0.3 (46)	0.4 (36)				
Bangladeshi	0.2 (102)	0.1 (<5)	0.1 (14)	0.2 (16)				
Other Asian	0.3 (166)	0.2 (11)	0.2 (29)	0.2 (17)				
Caribbean	0.6 (314)	0.3 (12)	0.5 (72)	0.4 (37)				
Black African	0.2 (123)	0.1 (<5)	0.3 (36)	0.2 (19)				
Other	0.6 (316)	0.4 (16)	0.6 (80)	0.5 (44)				
BMI recorded	97.0 (52299)	96.2 (4363)	95.7 (13013)	95.8 (8811)	96.9 (16143)	95.6 (959)	96.4 (2845)	95.7 (1342)
Mean BMI in kg/m2 (SD)	28.7 (5.7)	28.3 (5.5)	28.4 (5.6)	28.3 (5.6)	28.5 (5.8)	28.3 (6.0)	28.1 (5.8)	28.2 (5.7)
SBP recorded	100.0 (53912)	100.0 (4532)	100.0 (13594)	100.0 (9197)	99.8 (16636)	99.8 (1001)	99.8 (2945)	100.0 (1402)
Systolic blood pressure in mmHg (SD)	131.2 (17.5)	131.7 (17.1)	131.3 (17.1)	131.2 (17.2)	132.3 (17.2)	132.0 (16.0)	131.8 (16.9)	133.1 (17.2)
Smoking								
Recorded	99.9 (53856)	100.0 (4532)	99.9 (13578)	99.8 (9185)	99.9 (16653)	100.0 (1003)	99.9 (2948)	99.8 (1399)
Non-smoker	50.5 (27255)	52.1 (2360)	52.9 (7193)	53.4 (4910)	50.9 (8480)	53.2 (534)	52.6 (1552)	52.1 (730)

Ex-smoker	41.5 (22380)	40.8 (1852)	39.5 (5373)	39.0 (3591)	42.3 (7055)	40.4 (405)	40.0 (1179)	40.9 (574)
Current smoker	7.8 (4221)	7.1 (320)	7.4 (1012)	7.4 (684)	6.7 (1118)	6.4 (64)	7.4 (217)	6.8 (95)
Current light smoker	4.4 (2375)	3.9 (177)	4.3 (582)	4.3 (391)	4.1 (678)	4.3 (43)	4.7 (139)	5.1 (72)
Current moderate smoker	2.1 (1152)	1.9 (87)	1.9 (264)	1.8 (161)	1.7 (283)	1.5 (15)	1.6 (48)	1.2 (17)
Current heavy smoker	1.3 (694)	1.2 (56)	1.2 (166)	1.4 (132)	0.9 (157)	0.6 (6)	1.0 (30)	0.4 (6)
Not recorded	0.1 (65)	0.0 (2)	0.1 (19)	0.2 (14)	0.1 (11)	0	0.1 (2)	0.2 (3)
Alcohol consumption								
Recorded	94.7 (51063)	93.9 (4257)	94.2 (12815)	94.5 (8689)	94.9 (15814)	93.1 (934)	94.1 (2775)	93.6 (1312)
Non-drinker	34.2 (18465)	29.3 (1328)	33.7 (4583)	36.5 (3361)	32.4 (5400)	29.5 (296)	33.1 (976)	32.7 (459)
Trivial (< 1 unit per day)	28.5 (15364)	27.3 (1238)	26.8 (3642)	26.0 (2393)	32.9 (5484)	29.4 (295)	31.4 (926)	30.2 (424)
Light (1-2 units per day)	13.9 (7470)	14.6 (664)	13.4 (1823)	13.3 (1221)	17.9 (2975)	18.2 (183)	16.1 (476)	17.3 (243)
Moderate (3-6 units/day)	16.1 (8681)	19.9 (901)	17.9 (2433)	16.1 (1477)	6.3 (1054)	8.0 (80)	6.7 (197)	6.3 (89)
Heavy (7-9 units/day)	1.3 (712)	1.7 (79)	1.6 (222)	1.6 (149)	3.2 (530)	4.6 (46)	4.8 (143)	4.5 (63)
Very heavy (>9 units/day)	0.7 (371)	1.0 (47)	0.8 (112)	1.0 (88)	2.2 (371)	3.4 (34)	1.9 (57)	2.4 (34)
Not recorded	5.3 (2858)	6.1 (277)	5.8 (782)	5.5 (510)	5.1 (850)	6.9 (69)	5.9 (175)	6.4 (90)
Townsend quintiles								
1 (Affluent)	21.2 (11446)	25.5 (1154)	20.8 (2833)	22.3 (2054)	25.3 (4224)	28.3 (284)	28.1 (828)	29.3 (411)
2	21.3 (11506)	24.6 (1116)	22.1 (3009)	21.5 (1979)	25.8 (4294)	28.1 (282)	27.2 (802)	24.4 (342)
3	20.5 (11039)	20.3 (922)	21.7 (2948)	20.0 (1838)	21.8 (3636)	20.9 (210)	20.3 (598)	20.5 (287)
4	18.6 (10026)	17.2 (782)	19.5 (2648)	19.2 (1766)	17.4 (2899)	13.9 (139)	15.9 (469)	16.1 (226)
5 (Deprived)	18.4 (9904)	12.4 (560)	15.9 (2159)	17.0 (1562)	9.6 (1603)	8.8 (88)	8.6 (253)	9.7 (136)

eTable 4 ***Patients without atrial fibrillation****: Characteristics of patients in the QResearch and CPRD cohorts, percentage (numbers).*

	QResearch				CPRD			
	**warfarin**	**dabigatran**	**rivaroxaban**	**apixaban**	**warfarin**	**dabigatran**	**rivaroxaban**	**apixaban**
	% (n)	% (n)	% (n)	% (n)	% (n)	% (n)	% (n)	% (n)
Total N of patients	47331	1868	18423	7132	14315	339	2893	490
Age at baseline								
20-49	16.3 (7719)	6.6 (124)	14.2 (2619)	6.2 (444)	16.7 (2392)	5.6 (19)	16.5 (477)	5.7 (28)
50-59	13.8 (6532)	9.7 (181)	12.9 (2385)	8.2 (587)	13.9 (1989)	12.1 (41)	13.3 (385)	6.5 (32)
60-69	21.4 (10127)	22.1 (413)	20.5 (3785)	17.3 (1231)	21.0 (2999)	20.4 (69)	20.3 (588)	15.1 (74)
70-79	25.8 (12204)	32.6 (609)	25.3 (4660)	29.2 (2081)	25.9 (3703)	31.9 (108)	24.5 (709)	30.2 (148)
80-89	19.3 (9158)	24.3 (453)	21.3 (3919)	30.1 (2146)	19.1 (2731)	24.8 (84)	20.1 (582)	32.0 (157)
90 and over	3.4 (1591)	4.7 (88)	5.7 (1055)	9.0 (643)	3.5 (500)	5.3 (18)	5.3 (152)	10.4 (51)
Ethnicity recorded	82.8 (39209)	82.0 (1531)	80.3 (14787)	82.9 (5910)	97.0 (13885)	95.6 (324)	95.0 (2748)	96.3 (472)
White or not recorded (UK or European)	94.5 (44735)	96.8 (1808)	95.9 (17673)	96.2 (6864)	96.0 (13745)	98.5 (334)	96.9 (2804)	98.4 (482)
Ethnic minorities					4.0 (570)	1.5 (5)	3.1 (89)	1.6 (8)
Indian	1.0 (477)	0.9 (17)	0.7 (136)	0.8 (60)				
Pakistani	0.6 (292)	0.3 (5)	0.4 (82)	0.5 (36)				
Bangladeshi	0.3 (125)	0.2 (<5)	0.1 (20)	0.2 (16)				
Other Asian	0.4 (199)	0.3 (6)	0.3 (56)	0.2 (16)				
Caribbean	1.2 (568)	0.9 (17)	0.9 (175)	0.7 (52)				
Black African	0.8 (355)	0.2 (<5)	0.6 (114)	0.2 (16)				
Other	1.1 (537)	0.3 (6)	0.9 (157)	0.8 (60)				
BMI recorded	94.7 (44826)	95.0 (1774)	94.0 (17317)	95.4 (6803)	94.9 (13580)	95.6 (324)	94.5 (2734)	95.9 (470)
Mean BMI in kg/m2 (SD)	28.8 (5.8)	28.8 (5.6)	29.0 (5.9)	28.4 (5.7)	28.8 (6.1)	28.0 (5.1)	28.7 (6.2)	27.8 (5.9)
SBP recorded	99.5 (47115)	99.7 (1863)	99.3 (18294)	99.7 (7108)	98.9 (14160)	99.7 (338)	98.8 (2859)	99.6 (488)
Systolic blood pressure in mmHg (SD)	131.0 (17.8)	132.6 (18.0)	131.6 (17.3)	130.9 (18.0)	131.7 (17.0)	133.1 (16.2)	131.8 (16.4)	131.5 (16.9)
Smoking								
Recorded	99.6 (47142)	99.7 (1863)	99.6 (18353)	99.7 (7113)	99.9 (14300)	100.0 (339)	99.7 (2884)	99.6 (488)
Non-smoker	49.9 (23605)	49.9 (933)	51.9 (9563)	50.3 (3589)	49.2 (7049)	50.4 (171)	51.3 (1483)	50.2 (246)

Ex-smoker	35.5 (16803)	38.8 (725)	34.3 (6311)	38.6 (2755)	37.2 (5320)	40.1 (136)	33.9 (981)	42.4 (208)
Current smoker	14.2 (6734)	11.0 (205)	13.5 (2479)	10.8 (769)	13.5 (1931)	9.4 (32)	14.5 (420)	6.9 (34)
Current light smoker	7.1 (3382)	5.7 (107)	7.1 (1303)	6.2 (445)	7.4 (1056)	7.1 (24)	8.1 (233)	4.7 (23)
Current moderate smoker	4.2 (2001)	3.3 (62)	3.8 (697)	2.8 (198)	3.8 (547)	1.2 (4)	4.0 (117)	1.2 (6)
Current heavy smoker	2.9 (1351)	1.9 (36)	2.6 (479)	1.8 (126)	2.3 (328)	1.2 (4)	2.4 (70)	1.0 (5)
Not recorded	0.4 (189)	0.3 (5)	0.4 (70)	0.3 (19)	0.1 (15)	0	0.3 (9)	0.4 (2)
Alcohol consumption								
Recorded	91.5 (43321)	92.7 (1732)	90.9 (16753)	94.0 (6705)	92.0 (13163)	91.7 (311)	90.1 (2606)	92.9 (455)
Non-drinker	34.9 (16526)	31.8 (594)	34.5 (6354)	38.9 (2776)	33.5 (4799)	30.7 (104)	33.7 (974)	34.1 (167)
Trivial (< 1 unit per day)	28.0 (13241)	28.5 (532)	27.6 (5088)	26.0 (1854)	31.9 (4565)	33.3 (113)	30.5 (883)	33.5 (164)
Light (1-2 units per day)	12.4 (5860)	13.1 (245)	12.6 (2326)	12.8 (914)	15.6 (2226)	17.7 (60)	15.2 (439)	14.5 (71)
Moderate (3-6 units/day)	14.0 (6645)	16.8 (313)	14.0 (2571)	13.9 (993)	5.9 (845)	5.9 (20)	6.1 (177)	7.8 (38)
Heavy (7-9 units/day)	1.2 (588)	1.5 (28)	1.3 (231)	1.4 (100)	3.0 (423)	2.4 (8)	2.7 (77)	1.6 (8)
Very heavy (>9 units/day)	1.0 (461)	1.1 (20)	1.0 (183)	1.0 (68)	2.1 (305)	1.8 (6)	1.9 (56)	1.4 (7)
Not recorded	8.5 (4010)	7.3 (136)	9.1 (1670)	6.0 (427)	8.0 (1152)	8.3 (28)	9.9 (287)	7.1 (35)
Townsend quintiles								
1 (Affluent)	19.3 (9125)	24.0 (448)	19.7 (3622)	20.0 (1428)	23.3 (3334)	28.3 (96)	23.7 (687)	28.6 (140)
2	19.9 (9412)	20.3 (380)	20.5 (3771)	21.7 (1545)	23.5 (3362)	30.1 (102)	24.9 (720)	26.1 (128)
3	19.9 (9419)	22.7 (424)	21.6 (3982)	20.7 (1476)	21.5 (3071)	18.3 (62)	21.4 (619)	22.0 (108)
4	19.7 (9328)	17.1 (319)	20.7 (3816)	19.7 (1408)	19.5 (2786)	13.9 (47)	19.1 (553)	16.7 (82)
5 (Deprived)	21.2 (10047)	15.9 (297)	17.5 (3232)	17.9 (1275)	12.2 (1752)	9.4 (32)	10.8 (313)	6.5 (32)

*eTable 5 **All patients**: Rates per 1000py of outcomes by drug and adjusted hazard ratios with reference to warfarin in QResearch and CPRD*

	QResearch			CPRD		
	Person-years	Age-sex adjusted rate per 1000py (95%CI)	Adjusted hazard ratios (95%CI)[a]	Person-years	Age-sex adjusted rate per 1000py (95%CI)	Adjusted hazard ratios (95%CI)[a]
**Major bleeding**						
Warfarin	111,823	26.54 (25.58 to 27.50)	Reference	29,590	30.29 (28.30 to 32.28)	Reference
Dabigatran	6,117	22.92 (18.98 to 26.87)	0.91 (0.77 to 1.08)	1,069	20.24 (11.93 to 28.55)	0.74 (0.49 to 1.13)
Rivaroxaban	20,581	27.83 (25.54 to 30.12)	1.03 (0.93 to 1.14)	2,986	36.09 (29.16 to 43.01)	1.15 (0.91 to 1.44)
Apixaban	10,744	16.62 (14.14 to 19.10)	0.61 (0.52 to 0.71)*	987	22.29 (13.23 to 31.34)	0.75 (0.49 to 1.14)
**Intracranial bleed**						
Warfarin	113,705	6.21 (5.75 to 6.68)	Reference	30,033	6.39 (5.48 to 7.30)	Reference
Dabigatran	6,218	2.87 (1.53 to 4.20)	0.44 (0.27 to 0.71)*	1,078	1.73 (0.00 to 4.13)	n/a [b]
Rivaroxaban	20,823	4.49 (3.58 to 5.40)	0.68 (0.54 to 0.87)*	3,020	6.14 (3.32 to 8.95)	1.00 (0.59 to 1.69)
Apixaban	10,805	3.37 (2.30 to 4.44)	0.48 (0.34 to 0.68)*	994	3.40 (0.00 to 6.86)	n/a[b]
**Haematuria**						
Warfarin	112,791	8.29 (7.76 to 8.82)	Reference	29,844	8.96 (7.88 to 10.03)	Reference
Dabigatran	6,173	6.56 (4.51 to 8.61)	0.89 (0.65 to 1.23)	1,074	7.02 (2.14 to 11.91)	0.91 (0.45 to 1.86)
Rivaroxaban	20,730	8.40 (7.13 to 9.66)	1.15 (0.95 to 1.38)	3,003	10.94 (7.13 to 14.75)	1.51 (0.98 to 2.32)
Apixaban	10,789	4.54 (3.29 to 5.78)	0.69 (0.51 to 0.94)	991	7.20 (2.24 to 12.16)	1.17 (0.57 to 2.42)
**Haemoptysis**						
Warfarin	113,667	1.49 (1.27 to 1.72)	Reference	30,019	1.68 (1.22 to 2.15)	Reference
Dabigatran	6,204	1.47 (0.55 to 2.39)	1.18 (0.62 to 2.27)	1,078	0.97 (0.00 to 2.87)	n/a[b]
Rivaroxaban	20,820	1.64 (1.09 to 2.20)	1.09 (0.71 to 1.66)	3,021	2.16 (0.43 to 3.89)	1.37 (0.51 to 3.70)
Apixaban	10,811	0.45 (0.05 to 0.85)	0.32 (0.13 to 0.82)	994	0.83 (0.00 to 2.47)	n/a[b]
**All GI bleed**						
Warfarin	113,044	10.50 (9.90 to 11.10)	Reference	29,863	13.28 (11.97 to 14.59)	Reference
Dabigatran	6,180	12.19 (9.23 to 15.15)	1.20 (0.94 to 1.52)	1,074	10.41 (4.48 to 16.34)	0.88 (0.49 to 1.57)
Rivaroxaban	20,717	13.44 (11.86 to 15.02)	1.16 (1.00 to 1.35)	3,008	17.18 (12.44 to 21.92)	1.04 (0.75 to 1.44)
Apixaban	10,774	8.47 (6.64 to 10.30)	0.68 (0.54 to 0.85)*	991	10.79 (4.29 to 17.30)	0.61 (0.33 to 1.15)
**Upper GI bleed**						
Warfarin	113,143	9.36 (8.79 to 9.93)	Reference	29,886	11.96 (10.72 to 13.20)	Reference
Dabigatran	6,181	11.62 (8.72 to 14.53)	1.24 (0.97 to 1.59)	1,075	9.28 (3.78 to 14.79)	0.89 (0.48 to 1.64)
Rivaroxaban	20,728	12.58 (11.05 to 14.11)	1.18 (1.01 to 1.37)	3,008	16.84 (12.14 to 21.53)	1.12 (0.80 to 1.56)
Apixaban	10,779	7.92 (6.15 to 9.69)	0.68 (0.53 to 0.87)*	992	9.67 (3.55 to 15.79)	0.61 (0.31 to 1.17)

**Rectal bleed**						
Warfarin	113,685	1.23 (1.03 to 1.44)	Reference	30,030	1.44 (1.01 to 1.87)	Reference
Dabigatran	6,218	0.70 (0.08 to 1.31)	0.86 (0.35 to 2.12)	1,078	1.12 (0.00 to 3.33)	n/a[b]
Rivaroxaban	20,826	0.84 (0.45 to 1.24)	0.94 (0.54 to 1.64)	3,022	0.34 (0.00 to 1.00)	n/a[b]
Apixaban	10,808	0.62 (0.14 to 1.09)	0.74 (0.32 to 1.73)	993	1.10 (0.00 to 3.25)	n/a[b]
**Ischaemic stroke[c]**						
Warfarin	93,464	12.68 (11.95 to 13.41)	Reference	24,808	13.57 (12.11 to 15.02)	Reference
Dabigatran	4,499	16.27 (12.59 to 19.95)	1.28 (1.01 to 1.62)	759	12.71 (4.65 to 20.76)	0.88 (0.46 to 1.66)
Rivaroxaban	17,275	12.04 (10.41 to 13.68)	0.94 (0.80 to 1.11)	2,424	17.34 (12.05 to 22.64)	1.17 (0.81 to 1.69)
Apixaban	7,883	15.20 (12.52 to 17.88)	1.14 (0.93 to 1.41)	656	16.15 (6.90 to 25.40)	1.02 (0.55 to 1.89)
**VTE[d]**						
Warfarin	88,065	11.03 (10.34 to 11.72)	Reference	22,203	11.23 (9.83 to 12.62)	Reference
Dabigatran	5,976	2.60 (1.32 to 3.89)	0.20 (0.12 to 0.33)*	1,012	6.96 (1.80 to 12.12)	0.49 (0.23 to 1.05)
Rivaroxaban	15,993	46.96 (43.54 to 50.38)	1.51 (1.34 to 1.71)*	2,258	56.88 (46.70 to 67.06)	1.86 (1.41 to 2.46)*
Apixaban	9,978	12.33 (9.86 to 14.81)	0.46 (0.37 to 0.57)*	913	8.23 (2.38 to 14.09)	0.49 (0.24 to 1.02)
**Mortality**						
Warfarin	113,799	49.31 (47.99 to 50.63)	Reference	30,057	47.13 (44.64 to 49.61)	Reference
Dabigatran	6,220	47.43 (39.08 to 55.79)	1.02 (0.91 to 1.15)	1,078	44.90 (32.55 to 57.25)	1.16 (0.88 to 1.54)
Rivaroxaban	20,837	67.96 (64.49 to 71.43)	1.32 (1.24 to 1.41)*	3,022	73.89 (64.31 to 83.48)	1.45 (1.24 to 1.70)*
Apixaban	10,812	59.40 (55.03 to 63.77)	1.12 (1.03 to 1.22)	994	62.05 (47.47 to 76.64)	1.37 (1.06 to 1.76)

[a]) adjusted for age, sex, ethnicity, smoking, alcohol, Townsend quintile, BMI, SPB, falls&hipfracture, hip/knee ops, comorbidities (alcoholism, AF, treated hypertension, CKD, COPD, liver disease, CHD, CCF, any cancer, valvular, peptic ulcer), previous events (bleed, VTE, ischaemic stroke), medications at the baseline (macrolides, antiplatelets, anticonvulsant, corticosteroids, NSAIDs, statin, hormones), study year

[b]) insufficient data for analysis

[c]) based on patients without previous ischaemic stroke

[d]) based on patients without previous VTE

*) P-value<0.01

eTable 6 ***Patients with and without atrial fibrillation****: Adjusted hazard ratios (95% CI) for outcomes associated with exposure to study drugs compared with warfarin, by database*

	**With atrial fibrillation**				**Without atrial fibrillation**			
	**QResearch**		**CPRD**		**QResearch**		**CPRD**	
Comparison with warfarin	Adjusted HR (95%CI)[a]	P-value	Adjusted HR (95%CI)[a]	P-value	Adjusted HR (95%CI)[a]	P-value	Adjusted HR (95%CI)[a]	P-value
**Major bleeding**								
Dabigatran	0.90 (0.74 to 1.10)	0.3	0.68 (0.42 to 1.11)	0.1	0.97 (0.68 to 1.37)	0.8	1.04 (0.46 to 2.37)	0.9
Rivaroxaban	1.10 (0.96 to 1.25)	0.2	1.21 (0.91 to 1.63)	0.2	0.94 (0.80 to 1.10)	0.4	1.03 (0.72 to 1.50)	0.9
Apixaban	0.62 (0.50 to 0.75)	<0.001	0.91 (0.57 to 1.44)	0.7	0.60 (0.46 to 0.79)	<0.001	n/a[b]	
**Intracranial bleed**								
Dabigatran	0.45 (0.26 to 0.77)	0.004	n/a[b]		0.44 (0.16 to 1.19)	0.1	n/a[b]	
Rivaroxaban	0.78 (0.59 to 1.04)	0.09	1.39 (0.76 to 2.57)	0.3	0.54 (0.35 to 0.82)	0.004	n/a[b]	
Apixaban	0.40 (0.25 to 0.64)	<0.001	n/a[b]		0.63 (0.37 to 1.08)	0.09	n/a[b]	
**Haematuria**								
Dabigatran	0.94 (0.66 to 1.35)	0.7	1.01 (0.47 to 2.20)	1	0.78 (0.38 to 1.59)	0.5	n/a[b]	
Rivaroxaban	1.21 (0.95 to 1.54)	0.1	1.68 (0.98 to 2.87)	0.06	1.07 (0.79 to 1.45)	0.7	1.24 (0.60 to 2.56)	0.6
Apixaban	0.68 (0.46 to 1.00)	0.05	1.26 (0.54 to 2.90)	0.6	0.73 (0.45 to 1.20)	0.2	n/a[b]	
**All GI bleed**								
Dabigatran	1.16 (0.87 to 1.53)	0.3	0.72 (0.35 to 1.47)	0.4	1.36 (0.85 to 2.18)	0.2	n/a[b]	
Rivaroxaban	1.25 (1.03 to 1.52)	0.03	1.04 (0.68 to 1.60)	0.9	1.02 (0.81 to 1.28)	0.9	0.99 (0.60 to 1.65)	1
Apixaban	0.76 (0.57 to 1.02)	0.07	0.75 (0.38 to 1.48)	0.4	0.55 (0.37 to 0.83)	0.004	n/a[b]	
**Upper GI bleed**								
Dabigatran	1.25 (0.94 to 1.67)	0.1	0.69 (0.32 to 1.47)	0.3	1.25 (0.75 to 2.08)	0.4	n/a[b]	
Rivaroxaban	1.27 (1.04 to 1.56)	0.02	1.07 (0.68 to 1.66)	0.8	1.03 (0.81 to 1.30)	0.8	1.14 (0.68 to 1.90)	0.6
Apixaban	0.77 (0.57 to 1.04)	0.09	0.70 (0.34 to 1.44)	0.3	0.55 (0.36 to 0.83)	0.005	n/a[b]	
**Ischaemic stroke**[c]								
Dabigatran	1.18 (0.90 to 1.55)	0.2	0.74 (0.35 to 1.59)	0.4	1.76 (1.09 to 2.83)	0.02	n/a[b]	
Rivaroxaban	0.95 (0.77 to 1.16)	0.6	1.46 (0.96 to 2.23)	0.08	0.98 (0.74 to 1.29)	0.9	0.67 (0.31 to 1.43)	0.3
Apixaban	1.15 (0.90 to 1.49)	0.3	0.95 (0.46 to 1.96)	0.9	1.16 (0.80 to 1.68)	0.4	n/a[b]	
**VTE**[d]								
Dabigatran	0.41 (0.18 to 0.93)	0.03	n/a[b]		0.15 (0.08 to 0.29)	<0.001	0.58 (0.25 to 1.35)	0.2
Rivaroxaban	1.34 (0.94 to 1.91)	0.1	1.14 (0.56 to 2.30)		1.43 (1.25 to 1.63)	<0.001	1.92 (1.41 to 2.62)	<0.001

Apixaban	0.82 (0.48 to 1.39)	0.5	0.99 (0.36 to 2.69)		0.42 (0.33 to 0.53)	<0.001	n/a[b]	
**Mortality**								
Dabigatran	0.98 (0.85 to 1.13)	0.8	1.03 (0.74 to 1.43)	0.9	1.10 (0.87 to 1.39)	0.4	1.52 (0.89 to 2.62)	0.1
Rivaroxaban	1.19 (1.09 to 1.30)	<0.001	1.18 (0.94 to 1.49)	0.1	1.47 (1.33 to 1.62)	<0.001	1.76 (1.39 to 2.22)	<0.001
Apixaban	1.10 (0.98 to 1.23)	0.1	1.34 (0.99 to 1.82)	0.06	1.15 (1.00 to 1.33)	0.05	1.30 (0.81 to 2.09)	0.3

[a]) adjusted for age, sex, ethnicity, smoking, alcohol, Townsend quintile, BMI, SPB, falls & hip fracture, hip/knee ops, comorbidities (alcoholism, AF, treated hypertension, CKD, COPD, liver disease, CHD, CCF, any cancer, valvular, peptic ulcer), previous events (bleed, VTE, ischaemic stroke), medications at the baseline (macrolides, antiplatelets, anticonvulsant, corticosteroids, NSAIDs, statin, hormones), study year

[b]) insufficient data for analysis

[c]) based on patients without previous ischaemic stroke

[d]) based on patients without previous VTE

eTable 7 ***Patients with and without atrial fibrillation****: Adjusted hazard ratios (95% CI) for outcomes associated with exposure to study drugs compared with* ***apixaban****, by database*

	**With atrial fibrillation**				**Without atrial fibrillation**			
	**QResearch**		**CPRD**		**QResearch**		**CPRD**	
Comparison with apixaban	Adjusted HR (95%CI)	P-value	Adjusted HR (95%CI)	P-value	Adjusted HR (95%CI)	P-value	Adjusted HR (95%CI)	P-value
**Major bleeding**								
Dabigatran	1.47 (1.12 to 1.92)	0.006	0.75 (0.39 to 1.43)	0.4	1.60 (1.05 to 2.45)	0.03	n/a[b]	
Rivaroxaban	1.78 (1.44 to 2.20)	<0.001	1.34 (0.82 to 2.17)	0.2	1.55 (1.19 to 2.04)	0.001	n/a[b]	
Warfarin	1.62 (1.32 to 1.99)	<0.001	1.10 (0.69 to 1.75)	0.7	1.66 (1.27 to 2.16)	<0.001	n/a[b]	
**Intracranial bleed**								
Dabigatran	1.12 (0.57 to 2.22)	0.7	n/a[b]		n/a[b]		n/a[b]	
Rivaroxaban	1.94 (1.19 to 3.16)	0.008	n/a[b]		0.85 (0.47 to 1.53)	0.6	n/a[b]	
Warfarin	2.48 (1.57 to 3.94)	<0.001	n/a[b]		1.58 (0.93 to 2.69)	0.09	n/a[b]	
**Haematuria**								
Dabigatran	1.39 (0.84 to 2.29)	0.2	0.81 (0.27 to 2.40)	0.7	1.07 (0.46 to 2.46)	0.9	n/a[b]	
Rivaroxaban	1.78 (1.19 to 2.64)	0.005	1.33 (0.56 to 3.16)	0.5	1.46 (0.89 to 2.41)	0.1	n/a[b]	
Warfarin	1.47 (1.00 to 2.17)	0.05	0.80 (0.34 to 1.84)	0.6	1.37 (0.83 to 2.24)	0.2	n/a[b]	
**All GI bleed**								
Dabigatran	1.51 (1.03 to 2.21)	0.03	0.96 (0.37 to 2.49)	0.9	2.45 (1.36 to 4.39)	0.003	n/a[b]	
Rivaroxaban	1.64 (1.22 to 2.21)	0.001	1.39 (0.68 to 2.86)	0.4	1.84 (1.23 to 2.75)	0.003	n/a[b]	
Warfarin	1.31 (0.98 to 1.75)	0.07	1.34 (0.68 to 2.65)	0.4	1.78 (1.20 to 2.66)	0.005	n/a[b]	
**Upper GI bleed**								
Dabigatran	1.62 (1.10 to 2.38)	0.02	0.98 (0.36 to 2.69)	1	2.26 (1.21 to 4.22)	0.01	n/a[b]	
Rivaroxaban	1.65 (1.21 to 2.24)	0.001	1.52 (0.72 to 3.23)	0.3	1.86 (1.23 to 2.82)	0.003	n/a[b]	
Warfarin	1.29 (0.96 to 1.75)	0.09	1.43 (0.70 to 2.92)	0.3	1.79 (1.18 to 2.71)	0.006	n/a[b]	
**Ischaemic stroke[c]**								
Dabigatran	1.02 (0.72 to 1.45)	0.9	0.78 (0.28 to 2.14)	0.6	1.51 (0.87 to 2.65)	0.1	n/a[b]	
Rivaroxaban	0.82 (0.62 to 1.08)	0.2	1.53 (0.73 to 3.21)	0.3	0.84 (0.58 to 1.22)	0.4	n/a[b]	
Warfarin	0.87 (0.67 to 1.12)	0.3	1.05 (0.51 to 2.15)	0.9	0.86 (0.60 to 1.25)	0.4	n/a[b]	
**VTE[d]**								
Dabigatran	0.50 (0.20 to 1.29)	0.2	n/a[b]		0.36 (0.19 to 0.70)	0.003	n/a[b]	
Rivaroxaban	1.64 (0.96 to 2.80)	0.07	1.15 (0.40 to 3.29)	0.8	3.41 (2.72 to 4.26)	<0.001	n/a[b]	
Warfarin	1.22 (0.72 to 2.08)	0.5	1.01 (0.37 to 2.75)	1	2.38 (1.88 to 3.03)	<0.001	n/a[b]	

**Mortality**								
Dabigatran	0.90 (0.76 to 1.06)	0.2	0.77 (0.50 to 1.18)	0.2	0.96 (0.74 to 1.24)	0.8	1.17 (0.59 to 2.33)	0.7
Rivaroxaban	1.08 (0.96 to 1.22)	0.2	0.88 (0.64 to 1.22)	0.5	1.28 (1.12 to 1.46)	<0.001	1.35 (0.84 to 2.16)	0.2
Warfarin	0.91 (0.81 to 1.02)	0.1	0.75 (0.55 to 1.01)	0.06	0.87 (0.76 to 1.00)	0.06	0.77 (0.48 to 1.23)	0.3

[a]) adjusted for age, sex, ethnicity, smoking, alcohol, Townsend quintile, BMI, SPB, falls & hip fracture, hip/knee ops, comorbidities (alcoholism, AF, treated hypertension, CKD, COPD, liver disease, CHD, CCF, any cancer, valvular, peptic ulcer), previous events (bleed, VTE, ischaemic stroke), medications at the baseline (macrolides, antiplatelets, anticonvulsant, corticosteroids, NSAIDs, statin, hormones), study year

[b]) insufficient data for analysis

[c]) based on patients without previous ischaemic stroke

[d]) based on patients without previous VTE

eTable 8 Cause of death by drug and database, proportion of deceased patients on the drug

	QResearch			CPRD		
	Cause of death Bleed	Cause of death Stroke/ VTE	Cause of death Other causes	Cause of death Bleed	Cause of death Stroke/ VTE	Cause of death Other causes
**All patients**						
Warfarin	5%	5%	89%	4%	8%	88%
Dabigatran	2%	6%	92%	0	10%	90%
Rivaroxaban	2%	5%	94%	4%	9%	87%
Apixaban	1%	4%	95%	4%	13%	83%
**Patients with atrial fibrillation**						
Warfarin	5%	5%	90%	4%	5%	91%
Dabigatran	3%	7%	91%	0	8%	92%
Rivaroxaban	2%	5%	92%	5%	9%	86%
Apixaban	1%	4%	95%	5%	13%	82%
**Patients without atrial fibrillation**						
Warfarin	5%	6%	89%	4%	12%	84%
Dabigatran	3%	4%	95%	0	14%	86%
Rivaroxaban	1%	4%	95%	3%	8%	88%
Apixaban	1%	4%	95%	0	14%	86%

Note: The percentages do not always total 100% because a few patients had records of both bleeding and stroke/VTE as cause of death and also because of rounded numbers. Because of the low numbers of deaths only percentage is provided due to the Data Protection Act.

eTable 9 ***Patients with and without atrial fibrillation****: Cohort characteristics by database, percentage (number) of patients on lower and higher doses for DOAC's*

	**With atrial fibrillation**				**Without atrial fibrillation**			
	**QResearch**		**CPRD**		**QResearch**		**CPRD**	
	**Lower dose**	**Higher dose**	**Lower dose**	**Higher dose**	**Lower dose**	**Higher dose**	**Lower dose**	**Higher dose**
Total Number of patients	8090	19240	1644	3711	6694	20729	1305	2417
**Anticoagulant**								
Dabigatran	29.4 (2378)	11.2 (2156)	33.1 (544)	12.4 (459)	18.3 (1224)	3.1 (644)	18.1 (236)	4.3 (103)
Rivaroxaban	31.0 (2511)	57.6 (11086)	34.5 (568)	64.2 (2382)	45.7 (3056)	74.1 (15367)	66.1 (862)	84.0 (2031)
Apixaban	39.6 (3201)	31.2 (5998)	32.4 (532)	23.4 (870)	36.1 (2414)	22.8 (4718)	15.9 (207)	11.7 (283)
Median (interquartile range) for days of treatment	214 (79 to 458)	249 (90 to 473)	156 (60 to 332)	152 (63 to 319)	62 (47 to 1647)	101 (51 to 202)	58 (38 to 100)	99 (49 to 205)
**Gender**								
Men	42.0 (3396)	59.2 (11388)	44.4 (730)	60.7 (2252)	41.0 (2745)	52.7 (10921)	43.4 (566)	50.8 (1228)
Women	58.0 (4694)	40.8 (7852)	55.6 (914)	39.3 (1459)	59.0 (3949)	47.3 (9808)	56.6 (739)	49.2 (1189)
Mean age at baseline	82.9 (8.1)	72.9 (10.5)	81.9 (8.6)	73.0 (10.6)	76.0 (13.7)	67.9 (15.2)	71.6 (15.8)	66.6 (15.8)
BMI recorded	95.1 (7697)	96.1 (18490)	95.5 (1570)	96.4 (3576)	95.0 (6358)	94.2 (19536)	94.9 (1238)	94.7 (2290)
Mean BMI in kg/m2 (SD)	26.9 (5.3)	28.9 (5.6)	26.8 (5.7)	28.8 (5.8)	27.9 (5.6)	29.1 (5.9)	28.0 (5.9)	28.7 (6.2)
**Smoking**								
Non-smoker	56.3 (4556)	51.5 (9907)	56.1 (923)	51.0 (1893)	55.2 (3696)	50.1 (10389)	54.9 (716)	49.0 (1184)
Ex-smoker	38.3 (3098)	40.1 (7718)	38.4 (632)	41.1 (1526)	35.9 (2405)	35.6 (7386)	34.3 (448)	36.3 (877)
Current smoker	5.2 (422)	8.3 (1594)	5.4 (88)	7.8 (288)	8.6 (576)	13.9 (2877)	10.6 (138)	14.4 (348)
**Alcohol consumption**								
Recorded	93.6 (7571)	94.5 (18190)	93.1 (1531)	94.0 (3490)	92.5 (6194)	91.6 (18996)	90.7 (1184)	90.5 (2188)
Non-drinker	42.7 (3456)	30.2 (5816)	40.0 (657)	28.9 (1074)	40.4 (2707)	33.9 (7017)	36.2 (473)	31.9 (772)
Trivial (< 1 unit per day)	26.9 (2179)	26.5 (5094)	31.0 (509)	30.6 (1136)	27.5 (1841)	27.2 (5633)	31.5 (411)	31.0 (749)
Light (1-2 units per day)	11.5 (927)	14.5 (2781)	13.9 (229)	18.1 (673)	11.6 (775)	13.1 (2710)	13.6 (178)	16.2 (392)
Moderate (3-6 units/day)	11.4 (920)	20.2 (3891)	4.2 (69)	8.0 (297)	11.6 (779)	14.9 (3098)	6.2 (81)	6.4 (154)
Heavy (7-9 units/day)	0.7 (58)	2.0 (392)	3.0 (49)	5.5 (203)	0.8 (55)	1.5 (304)	1.7 (22)	2.9 (71)

Very heavy (>9 units/day)	0.4 (31)	1.1 (216)	1.1 (18)	2.9 (107)	0.6 (37)	1.1 (234)	1.5 (19)	2.1 (50)
Not recorded	6.4 (519)	5.5 (1050)	6.9 (113)	6.0 (221)	7.5 (500)	8.4 (1733)	9.3 (121)	9.5 (229)
**Townsend quintiles**								
1 (Affluent)	20.9 (1693)	22.6 (4348)	27.1 (446)	29.0 (1077)	20.3 (1358)	20.0 (4140)	26.9 (351)	23.7 (572)
2	20.8 (1684)	23.0 (4420)	25.5 (419)	27.1 (1007)	20.6 (1380)	20.8 (4316)	25.5 (333)	25.5 (617)
3	20.9 (1691)	20.9 (4017)	19.9 (327)	20.7 (768)	21.3 (1429)	21.5 (4453)	21.1 (275)	21.3 (514)
4	20.5 (1662)	18.4 (3534)	16.7 (274)	15.1 (560)	19.7 (1317)	20.4 (4226)	17.9 (233)	18.6 (449)
5 (Deprived)	16.8 (1360)	15.2 (2921)	10.8 (178)	8.1 (299)	18.1 (1210)	17.3 (3594)	8.7 (113)	10.9 (264)

eTable 10 ***Patients with and without atrial fibrillation****: Comorbidities, previous events and other medications, percentage (number) of patients on lower and higher doses for DOAC's.*

	With atrial fibrillation				Without atrial fibrillation			
	QResearch		CPRD		QResearch		CPRD	
Total N	**Lower dose**	**Higher dose**	**Lower dose**	**Higher dose**	**Lower dose**	**Higher dose**	**Lower dose**	**Higher dose**
**Comorbidities at baseline**								
Alcohol dependence	2.1 (171)	3.4 (646)	1.9 (32)	3.4 (128)	2.2 (146)	3.6 (738)	2.5 (32)	3.6 (88)
Bleeding disorders	1.2 (101)	0.9 (175)	1.8 (30)	1.2 (43)	1.2 (82)	1.1 (226)	1.5 (20)	1.7 (41)
Cancer (any)	15.4 (1245)	12.1 (2333)	14.1 (231)	11.9 (442)	14.5 (968)	12.5 (2591)	14.1 (184)	13.3 (321)
Chronic liver disease or pancreatitis	1.5 (122)	1.3 (247)	1.3 (22)	1.5 (54)	1.2 (82)	1.3 (264)	1.3 (17)	1.7 (42)
Chronic obstructive pulmonary disease	11.2 (906)	8.9 (1710)	10.1 (166)	8.9 (331)	8.5 (568)	8.5 (1754)	6.9 (90)	8.6 (209)
Chronic renal disease	4.2 (336)	0.7 (128)	4.1 (68)	0.9 (34)	3.1 (209)	1.0 (213)	3.3 (43)	1.1 (26)
Congestive cardiac failure	16.7 (1355)	9.7 (1873)	17.0 (280)	9.8 (364)	9.1 (608)	5.1 (1060)	6.5 (85)	5.5 (132)
Coronary heart disease	28.9 (2335)	20.3 (3901)	27.4 (451)	20.3 (754)	20.8 (1393)	14.7 (3053)	17.6 (230)	15.0 (363)
Diabetes	20.1 (1629)	17.5 (3362)	19.9 (327)	17.2 (637)	18.0 (1208)	16.0 (3315)	16.2 (212)	13.8 (333)
Dyspepsia	19.2 (1557)	18.4 (3542)	27.3 (449)	25.6 (949)	17.8 (1193)	17.3 (3588)	26.4 (345)	24.3 (588)
Falls or hip fracture (within 180 days)	12.3 (995)	5.7 (1098)	9.5 (157)	4.9 (183)	17.9 (1195)	5.5 (1132)	7.9 (103)	4.9 (118)
Hip or knee operation (within 180 days)	1.3 (102)	0.7 (128)	2.4 (39)	1.3 (49)	22.6 (1513)	2.4 (496)	21.9 (286)	3.6 (88)
Hypertension	66.2 (5354)	57.0 (10958)	69.6 (1144)	58.5 (2171)	52.2 (3497)	41.2 (8539)	49.5 (646)	42.0 (1014)
Ischaemic stroke*	26.1 (2115)	17.0 (3265)	29.1 (478)	19.8 (736)	19.0 (1272)	12.9 (2680)	15.6 (204)	14.4 (348)
Oesophageal varices	0.1 (9)	0.1 (19)	<5	<5	0.1 (4)	0.1 (24)	<5	<5
Peptic ulcer	9.4 (757)	6.9 (1322)	10.0 (164)	8.4 (310)	7.3 (486)	5.8 (1198)	7.8 (102)	6.5 (158)
Valvular heart disease	12.6 (1022)	8.2 (1572)	9.9 (162)	6.3 (233)	7.0 (471)	4.5 (934)	4.8 (63)	3.9 (94)
Venous thromboembolism*	5.1 (412)	4.9 (940)	8.3 (137)	5.9 (219)	13.2 (883)	37.4 (7747)	27.3 (356)	46.0 (1112)
**Previous bleed (any)***	29.4 (2375)	25.1 (4835)	31.7 (521)	28.6 (1061)	24.8 (1659)	23.0 (4763)	27.8 (363)	26.5 (641)
Previous Intracranial bleed*	1.8 (142)	1.0 (201)	2.4 (40)	1.3 (50)	1.4 (94)	1.1 (221)	2.1 (28)	1.4 (35)
Previous Haematuria	13.9 (1122)	11.6 (2236)	13.7 (226)	11.0 (408)	10.0 (668)	9.7 (2009)	9.6 (125)	9.5 (229)
Previous Haemoptysis*	2.5 (203)	2.7 (514)	2.9 (48)	3.4 (128)	2.4 (158)	2.7 (551)	3.8 (50)	4.1 (100)
Previous All GI bleed*	15.5 (1255)	13.5 (2598)	18.2 (299)	16.9 (629)	14.2 (952)	12.7 (2636)	16.2 (211)	15.2 (367)
Previous upper GI bleed*	6.1 (491)	4.3 (823)	6.8 (111)	5.6 (208)	5.1 (341)	4.0 (837)	6.2 (81)	5.8 (141)

Previous Rectal bleed*	10.9 (882)	10.3 (1988)	13.4 (220)	13.1 (485)	10.4 (698)	9.8 (2029)	12.3 (161)	11.1 (268)
**Other medications**								
Proton Pump Inhibitors	48.0 (3882)	40.4 (7764)	45.3 (745)	40.6 (1506)	47.6 (3185)	39.2 (8132)	44.3 (578)	40.7 (983)
Antibiotics#	9.1 (735)	6.7 (1298)	7.6 (125)	6.6 (246)	8.4 (560)	7.6 (1575)	8.4 (110)	9.5 (230)
Antiplatelet	24.0 (1943)	17.7 (3408)	39.6 (651)	30.3 (1124)	20.5 (1374)	16.0 (3316)	23.7 (309)	18.0 (434)
Antidepressants	17.8 (1443)	14.9 (2871)	18.0 (296)	15.7 (583)	21.0 (1409)	21.5 (4466)	23.5 (307)	22.2 (536)
Anticonvulsants	0.7 (56)	0.6 (118)	0.7 (11)	0.9 (32)	0.9 (62)	1.2 (242)	0.9 (12)	1.4 (34)
NSAIDs	5.5 (447)	7.6 (1456)	5.1 (84)	7.5 (279)	16.1 (1076)	10.1 (2085)	17.9 (234)	10.1 (245)
Corticosteroids	12.2 (986)	9.8 (1884)	10.5 (172)	9.1 (337)	10.1 (678)	10.1 (2098)	10.6 (138)	11.7 (282)
Statins	52.7 (4265)	52.5 (10110)	53.0 (872)	52.6 (1953)	44.6 (2987)	39.0 (8092)	39.8 (519)	36.7 (888)
Hormones (women)	1.0 (46)	1.9 (147)	1.2 (11)	3.8 (55)	2.1 (84)	2.6 (251)	5.8 (43)	8.7 (104)

*) Based on GP and HES records

#) In the last 6 months prior to the drug start date

eTable 11 ***Patients with atrial fibrillation****: Rates per 1000py of outcomes by drug and dose for DOAC's and adjusted hazard rations with reference to* ***warfarin*** *in QResearch and CPRD*

	QResearch				CPRD			
With reference to warfarin	Person-years	N of events	Age-sex adjusted rate per 1000py (95%CI)	Adjusted hazard ratios (95%CI)[a]	Person-years	N of events	Age-sex adjusted rate per 1000py (95%CI)	Adjusted hazard ratios (95%CI)[a]
**Major bleeding**								
Dabigatran <300	2422	67	25.4 (18.0 to 32.7)	0.97 (0.76 to 1.24)	483	12	26.6 (7.5 to 45.8)	0.74 (0.41 to 1.32)
Dabigatran 300+	2360	36	25.9 (2.5 to 49.4)	0.79 (0.56 to 1.10)	346	<5	5.7 (0.0 to 12.2)	n/a
Rivaroxaban <20	2046	79	36.0 (24.0 to 48.1)	1.26 (1.00 to 1.60)	335	15	42.7 (14.6 to 70.8)	1.18 (0.69 to 2.02)
Rivaroxaban 20+	9836	242	25.2 (22.0 to 28.4)	1.05 (0.91 to 1.22)	1439	43	31.9 (22.3 to 41.5)	1.08 (0.77 to 1.52)
Apixaban <10	2396	50	28.4 (13.3 to 43.5)	0.67 (0.50 to 0.91)*	267	8	48.5 (0.0 to 102.7)	0.73 (0.36 to 1.52)
Apixaban 10+	4749	62	13.4 (9.9 to 17.0)	0.56 (0.43 to 0.73)*	477	13	31.0 (12.8 to 49.2)	1.02 (0.57 to 1.82)
**Intracranial bleed**								
Dabigatran <300	2465	10	3.3 (1.0 to 5.6)	0.48 (0.26 to 0.91)	489	0	-	n/a[b]
Dabigatran 300+	2400	<5	7.6 (0.0 to 20.3)	n/a[b]	348	<5	1.4 (0.0 to 4.2)	n/a[b]
Rivaroxaban <20	2077	17	7.8 (3.3 to 12.3)	0.86 (0.52 to 1.42)	339	<5	12.3 (0.0 to 26.0)	n/a[b]
Rivaroxaban 20+	9946	47	5.0 (3.6 to 6.5)	0.79 (0.57 to 1.09)	1453	9	6.6 (2.2 to 10.9)	1.24 (0.59 to 2.61)
Apixaban <10	2408	11	2.8 (0.0 to 5.6)	0.44 (0.23 to 0.82)*	268	0	-	n/a[b]
Apixaban 10+	4769	11	2.8 (1.0 to 4.5)	0.41 (0.22 to 0.76)*	481	<5	10.3 (0.0 to 21.7)	n/a[b]
**Haematuria**								
Dabigatran <300	2439	19	7.9 (3.5 to 12.3)	1.04 (0.65 to 1.65)	486	5	15.8 (0.0 to 33.0)	1.26 (0.51 to 3.12)
Dabigatran 300+	2391	11	2.7 (1.1 to 4.4)	0.71 (0.39 to 1.30)	348	0	-	n/a[b]
Rivaroxaban <20	2060	23	14.4 (5.7 to 23.1)	1.64 (1.06 to 2.55)	336	<5	8.9 (0.1 to 17.8)	n/a[b]
Rivaroxaban 20+	9910	72	7.3 (5.6 to 9.1)	1.10 (0.83 to 1.44)	1447	15	10.8 (5.3 to 16.3)	1.57 (0.86 to 2.88)
Apixaban <10	2405	13	8.4 (0.0 to 17.1)	0.84 (0.47 to 1.50)	267	<5	18.5 (0.0 to 49.0)	n/a[b]
Apixaban 10+	4765	16	3.3 (1.7 to 5.0)	0.51 (0.30 to 0.85)	480	<5	5.3 (0.0 to 11.2)	n/a[b]
**All GI bleed**								
Dabigatran <300	2449	38	13.9 (8.6 to 19.1)	1.37 (0.98 to 1.91)	487	6	8.4 (1.4 to 15.4)	0.83 (0.37 to 1.89)
Dabigatran 300+	2385	15	13.6 (0.0 to 33.1)	0.86 (0.51 to 1.44)	346	<5	4.3 (0.0 to 10.2)	n/a[b]
Rivaroxaban <20	2068	38	13.0 (6.3 to 19.7)	1.38 (0.98 to 1.95)	338	8	23.5 (0.2 to 46.8)	1.17 (0.56 to 2.46)
Rivaroxaban 20+	9893	110	11.4 (9.3 to 13.5)	1.20 (0.96 to 1.50)	1447	18	13.5 (7.2 to 19.8)	0.86 (0.51 to 1.44)
Apixaban <10	2404	23	12.8 (3.6 to 21.9)	0.70 (0.45 to 1.09)	268	<5	28.1 (0.0 to 72.7)	n/a[b]
Apixaban 10+	4755	36	7.6 (4.9 to 10.2)	0.81 (0.56 to 1.16)	479	6	15.3 (2.4 to 28.1)	0.85 (0.36 to 1.99)
**Upper GI bleed**								

Dabigatran <300	2449	38	13.9 (8.6 to 19.1)	1.49 (1.06 to 2.08)	487	6	8.4 (1.4 to 15.4)	0.91 (0.40 to 2.06)
Dabigatran 300+	2385	14	13.4 (0.0 to 32.9)	0.90 (0.53 to 1.54)	347	<5	2.0 (0.0 to 5.8)	n/a[b]
Rivaroxaban <20	2070	35	12.4 (5.8 to 19.0)	1.34 (0.94 to 1.92)	338	8	23.5 (0.2 to 46.8)	1.23 (0.59 to 2.60)
Rivaroxaban 20+	9900	104	10.8 (8.7 to 12.9)	1.23 (0.98 to 1.55)	1447	17	12.7 (6.6 to 18.8)	0.86 (0.50 to 1.47)
Apixaban <10	2404	21	11.4 (2.6 to 20.1)	0.68 (0.43 to 1.08)	268	<5	28.1 (0.0 to 72.7)	n/a[b]
Apixaban 10+	4757	34	7.2 (4.6 to 9.8)	0.83 (0.57 to 1.21)	480	5	13.2 (1.0 to 25.4)	0.75 (0.29 to 1.88)
**Ischaemic stroke[c]**								
Dabigatran <300	1686	32	15.8 (9.3 to 22.3)	1.11 (0.77 to 1.59)	327	5	12.2 (0.4 to 23.9)	0.82 (0.34 to 2.02)
Dabigatran 300+	1889	26	10.5 (6.3 to 14.8)	1.37 (0.92 to 2.05)	276	<5	3.7 (0.0 to 9.3)	n/a[b]
Rivaroxaban <20	1560	32	17.9 (9.2 to 26.7)	1.13 (0.78 to 1.63)	231	10	32.7 (10.1 to 55.3)	1.87 (0.95 to 3.69)
Rivaroxaban 20+	8226	91	11.5 (9.1 to 13.9)	0.90 (0.71 to 1.14)	1147	23	20.4 (11.9 to 28.8)	1.33 (0.82 to 2.16)
Apixaban <10	1675	36	19.6 (7.7 to 31.4)	1.16 (0.81 to 1.67)	174	<5	17.9 (0.0 to 41.3)	n/a[b]
Apixaban 10+	3677	45	11.9 (8.3 to 15.6)	1.09 (0.78 to 1.51)	340	5	21.1 (0.4 to 41.9)	0.97 (0.38 to 2.46)
**VTE[d]**								
Dabigatran <300	2355	<5	2.2 (0.0 to 5.5)	n/a[b]	461	0	-	n/a[b]
Dabigatran 300+	2352	<5	0.8 (0.0 to 1.8)	n/a[b]	329	<5	3.3 (0.0 to 9.9)	n/a[b]
Rivaroxaban <20	1961	7	2.5 (0.3 to 4.7)	0.80 (0.35 to 1.87)	304	<5	3.5 (0.0 to 8.6)	n/a[b]
Rivaroxaban 20+	9444	41	4.7 (3.2 to 6.1)	1.14 (0.73 to 1.79)	1357	9	7.2 (2.5 to 12.0)	1.10 (0.50 to 2.40)
Apixaban <10	2312	8	5.6 (0.0 to 13.3)	0.86 (0.38 to 1.95)	249	<5	1	n/a[b]
Apixaban 10+	4606	10	2.4 (0.8 to 4.1)	0.69 (0.32 to 1.47)	453	<5	6.0 (0.0 to 12.8)	n/a[b]
**Mortality**								
Dabigatran <300	2466	165	45.6 (37.6 to 53.7)	1.03 (0.88 to 1.21)	489	25	47.8 (25.9 to 69.8)	0.90 (0.60 to 1.35)
Dabigatran 300+	2401	39	74.9 (3.3 to 146.4)	0.78 (0.57 to 1.08)	348	11	23.3 (8.3 to 38.2)	1.50 (0.82 to 2.74)
Rivaroxaban <20	2078	251	91.2 (64.9 to 117.5)	1.28 (1.11 to 1.47)*	339	40	77.1 (48.3 to 105.8)	1.41 (1.00 to 1.98)
Rivaroxaban 20+	9953	440	47.0 (42.6 to 51.4)	1.11 (1.00 to 1.24)	1454	62	43.7 (32.7 to 54.8)	1.00 (0.75 to 1.33)
Apixaban <10	2410	292	80.2 (64.8 to 95.6)	1.23 (1.08 to 1.41)*	268	37	104.3 (51.6 to 157.0)	1.62 (1.13 to 2.33)*
Apixaban 10+	4770	167	43.8 (36.8 to 50.8)	0.98 (0.83 to 1.15)	481	18	45.9 (23.3 to 68.5)	0.99 (0.61 to 1.61)

[a]) adjusted for age, sex, ethnicity, smoking, alcohol, Townsend quintile, BMI, SPB, falls&hipfracture, hip/knee ops, comorbidities (alcoholism, AF, treated hypertension, CKD, COPD, liver disease, CHD, CCF, any cancer, valvular, peptic ulcer), previous events (bleed, VTE, ischaemic stroke), medications at the baseline (macrolides, antiplatelets, anticonvulsant, corticosteroids, NSAIDs, statin, hormones), study year

[b]) insufficient data for analysis

[c]) based on patients without previous ischaemic stroke

[d]) based on patients without previous VTE

*) P-value<0.01

*eTable 12 **Patients without atrial fibrillation**: Rates per 1000py of outcomes by drug and dose for DOAC's and adjusted hazard rations with reference to **warfarin** in QResearch and CPRD*

	QResearch				CPRD			
	Person-years	N of events	Age-sex adjusted rate per 1000py (95%CI)	Adjusted hazard ratios (95%CI)[a]	Person-years	N of events	Age-sex adjusted rate per 1000py (95%CI)	Adjusted hazard ratios (95%CI)[a]
**Major bleeding**								
Dabigatran <300	609	24	38.0 (12.9 to 63.0)	1.16 (0.77 to 1.75)	97	<5	4.4 (0.0 to 13.1)	n/a[b]
Dabigatran 300+	487	9	26.1 (0.0 to 53.5)	0.73 (0.38 to 1.41)	72	5	115.0 (0.0 to 236.1)	3.00 (1.21 to 7.41)
Rivaroxaban <20	996	39	37.9 (24.1 to 51.7)	1.03 (0.74 to 1.44)	201	11	44.1 (14.6 to 73.6)	1.34 (0.72 to 2.50)
Rivaroxaban 20+	6750	188	28.2 (24.1 to 32.2)	0.91 (0.76 to 1.09)	841	25	29.7 (18.0 to 41.5)	0.89 (0.57 to 1.40)
Apixaban <10	1044	36	22.6 (10.6 to 34.5)	0.82 (0.57 to 1.17)	76	<5	7.4 (0.0 to 18.9)	n/a[b]
Apixaban 10+	2083	34	15.7 (10.1 to 21.3)	0.49 (0.34 to 0.71)*	133	<5	7.0 (0.0 to 20.8)	n/a[b]
**Intracranial bleed**								
Dabigatran <300	615	<5	3.9 (0.0 to 8.2)	n/a[b]	97	<5	4.4 (0.0 to 13.1)	n/a[b]
Dabigatran 300+	489	0	-	n/a[b]	73	0	-	n/a[b]
Rivaroxaban <20	1003	11	13.7 (5.1 to 22.3)	1.27 (0.67 to 2.40)	203	<5	3.9 (0.0 to 9.5)	n/a[b]
Rivaroxaban 20+	6825	18	2.7 (1.4 to 3.9)	0.42 (0.25 to 0.70)*	850	<5	2.0 (0.0 to 4.9)	n/a[b]
Apixaban <10	1056	11	5.5 (1.3 to 9.7)	0.92 (0.47 to 1.78)	76	0	-	n/a[b]
Apixaban 10+	2094	8	3.6 (1.1 to 6.2)	0.49 (0.23 to 1.03)	133	0	-	n/a[b]
**Haematuria**								
Dabigatran <300	612	<5	5.6 (0.0 to 11.4)	n/a[b]	98	0	-	n/a[b]
Dabigatran 300+	488	<5	18.6 (0.0 to 45.3)	n/a[b]	73	<5	13.8 (0.0 to 40.7)	n/a[b]
Rivaroxaban <20	1001	13	14.3 (5.9 to 22.6)	1.42 (0.79 to 2.54)	201	<5	12.1 (0.2 to 23.9)	n/a[b]
Rivaroxaban 20+	6795	53	8.0 (5.8 to 10.2)	0.97 (0.69 to 1.35)	847	5	6.3 (0.7 to 11.9)	0.80 (0.30 to 2.14)
Apixaban <10	1054	9	5.6 (0.7 to 10.4)	0.95 (0.47 to 1.93)	76	<5	1.8 (0.0 to 5.2)	n/a[b]
Apixaban 10+	2091	11	4.4 (1.8 to 7.1)	0.62 (0.32 to 1.18)	133	<5	7.0 (0.0 to 20.7)	n/a[b]
**All GI bleed**								
Dabigatran <300	612	14	24.0 (1.0 to 46.9)	1.59 (0.92 to 2.74)	98	0	-	n/a[b]
Dabigatran 300+	488	5	7.4 (0.7 to 14.0)	1.05 (0.43 to 2.57)	72	<5	101.6 (0.0 to 220.3)	n/a[b]
Rivaroxaban <20	1001	14	8.6 (2.8 to 14.4)	0.75 (0.43 to 1.30)	202	<5	12.2 (0.0 to 26.6)	n/a[b]
Rivaroxaban 20+	6790	107	15.9 (12.9 to 18.9)	1.08 (0.85 to 1.37)	844	16	18.9 (9.5 to 28.2)	1.01 (0.57 to 1.80)
Apixaban <10	1049	15	9.6 (0.1 to 19.1)	0.68 (0.39 to 1.17)	76	<5	5.6 (0.0 to 16.5)	n/a[b]
Apixaban 10+	2091	16	8.0 (3.7 to 12.3)	0.51 (0.30 to 0.86)	133	0	-	n/a[b]
**Upper GI bleed**								

Dabigatran <300	613	12	21.5 (0.0 to 44.2)	1.48 (0.82 to 2.66)	98	0	-	n/a[b]
Dabigatran 300+	488	<5	6.3 (0.0 to 12.5)	n/a[b]	72	<5	101.7 (0.0 to 220.3)	n/a[b]
Rivaroxaban <20	1001	14	8.6 (2.8 to 14.4)	0.81 (0.47 to 1.40)	202	<5	12.1 (0.0 to 26.6)	n/a[b]
Rivaroxaban 20+	6793	98	14.6 (11.7 to 17.4)	1.07 (0.83 to 1.38)	844	16	18.9 (9.5 to 28.2)	1.16 (0.65 to 2.08)
Apixaban <10	1049	14	9.3 (0.0 to 18.8)	0.67 (0.38 to 1.18)	76	<5	5.6 (0.0 to 16.5)	n/a[b]
Apixaban 10+	2093	15	7.4 (3.3 to 11.6)	0.51 (0.29 to 0.87)	133	0	-	n/a[b]
**Ischaemic stroke[c]**								
Dabigatran <300	408	11	19.0 (6.8 to 31.2)	1.60 (0.86 to 2.96)	58	<5	40.5 (0.0 to 95.1)	n/a[b]
Dabigatran 300+	321	8	26.7 (0.2 to 53.2)	2.19 (1.08 to 4.47)	50	0	-	n/a[b]
Rivaroxaban <20	778	17	20.1 (8.7 to 31.5)	1.21 (0.72 to 2.02)	166	<5	7.0 (0.0 to 16.8)	n/a[b]
Rivaroxaban 20+	5957	60	10.2 (7.6 to 12.8)	0.87 (0.64 to 1.19)	739	6	7.6 (1.4 to 13.8)	0.63 (0.26 to 1.53)
Apixaban <10	708	20	13.6 (5.8 to 21.5)	1.26 (0.77 to 2.07)	44	<5	9.4 (0.0 to 27.7)	n/a[b]
Apixaban 10+	1499	23	13.7 (8.0 to 19.4)	1.08 (0.68 to 1.72)	71	<5	24.9 (0.0 to 60.4)	n/a[b]
**VTE[d]**								
Dabigatran <300	578	6	8.4 (1.1 to 15.7)	0.10 (0.05 to 0.23)*	89	<5	46.7 (0.0 to 104.6)	n/a[b]
Dabigatran 300+	447	<5	6.8 (0.0 to 13.7)	n/a[b]	69	<5	23.5 (0.0 to 57.2)	n/a[b]
Rivaroxaban <20	801	25	40.4 (22.2 to 58.5)	0.18 (0.12 to 0.28)*	136	28	279.2 (166.2 to 392.2)	1.31 (0.83 to 2.06)
Rivaroxaban 20+	3062	657	219.2 (202.3 to 236.1)	1.85 (1.61 to 2.11)*	370	81	258.6 (192.0 to 325.3)	2.19 (1.56 to 3.07)*
Apixaban <10	922	14	20.8 (3.7 to 37.9)	0.19 (0.11 to 0.32)*	67	<5	16.2 (0.0 to 38.8)	n/a[b]
Apixaban 10+	1722	74	52.8 (39.8 to 65.9)	0.58 (0.45 to 0.75)*	112	<5	8.1 (0.0 to 24.0)	n/a[b]
**Mortality**								
Dabigatran <300	615	61	80.2 (44.1 to 116.3)	1.26 (0.97 to 1.63)	98	13	121.8 (27.3 to 216.2)	1.92 (1.09 to 3.38)
Dabigatran 300+	489	12	76.0 (0.0 to 164.3)	0.67 (0.38 to 1.18)	73	<5	8.7 (0.0 to 25.9)	n/a[b]
Rivaroxaban <20	1004	164	115.9 (93.4 to 138.5)	1.67 (1.41 to 1.98)*	203	41	173.1 (112.0 to 234.1)	2.39 (1.69 to 3.38)*
Rivaroxaban 20+	6827	546	80.9 (74.1 to 87.7)	1.39 (1.24 to 1.55)*	850	75	90.6 (69.8 to 111.3)	1.46 (1.11 to 1.93)*
Apixaban <10	1057	175	119.6 (88.2 to 151.0)	1.33 (1.12 to 1.58)*	76	12	94.2 (27.7 to 160.8)	1.42 (0.78 to 2.59)
Apixaban 10+	2097	128	56.9 (46.7 to 67.1)	0.99 (0.81 to 1.20)	133	8	84.3 (0.0 to 174.7)	1.14 (0.55 to 2.35)

[a]) adjusted for age, sex, ethnicity, smoking, alcohol, Townsend quintile, BMI, SPB, falls&hipfracture, hip/knee ops, comorbidities (alcoholism, AF, treated hypertension, CKD, COPD, liver disease, CHD, CCF, any cancer, valvular, peptic ulcer), previous events (bleed, VTE, ischaemic stroke), medications at the baseline (macrolides, antiplatelets, anticonvulsant, corticosteroids, NSAIDs, statin, hormones), study year

[b]) insufficient data for analysis

[c]) based on patients without previous ischaemic stroke

[d]) based on patients without previous VTE

*) P-value<0.01

eTable 13 ***All patients****: Rates per 1000py of outcomes by drug and dose for DOAC's and adjusted hazard ratios with reference to warfarin in QResearch and CPRD*

	QResearch			CPRD		
	Person-years	Age-sex adjusted rate per 1000py (95%CI)	Adjusted hazard ratios (95%CI)[a]	Person-years	Age-sex adjusted rate per 1000py (95%CI)	Adjusted hazard ratios (95%CI)[a]
**Major bleeding**						
Dabigatran <300	3031	27.6 (19.8 to 35.3)	1.01 (0.81 to 1.24)	581	22.1 (6.1 to 38.0)	0.66 (0.38 to 1.15)
Dabigatran 300+	2847	25.6 (7.1 to 44.0)	0.77 (0.57 to 1.04)	418	54.0 (0.0 to 116.4)	0.84 (0.42 to 1.69)
Rivaroxaban <20	3043	35.9 (27.6 to 44.2)	1.17 (0.96 to 1.41)	536	40.1 (21.9 to 58.2)	1.26 (0.84 to 1.89)
Rivaroxaban 20+	16587	26.6 (24.1 to 29.1)	0.99 (0.89 to 1.11)	2280	31.3 (23.8 to 38.8)	1.01 (0.77 to 1.32)
Apixaban <10	3440	25.8 (15.8 to 35.7)	0.72 (0.58 to 0.91)*	343	34.6 (0.0 to 70.6)	0.69 (0.37 to 1.32)
Apixaban 10+	6832	14.2 (11.3 to 17.2)	0.53 (0.43 to 0.66)*	610	24.1 (10.6 to 37.6)	0.78 (0.45 to 1.35)
**Intracranial bleed**						
Dabigatran <300	3080	3.3 (1.3 to 5.3)	0.53 (0.31 to 0.90)	586	0.9 (0.0 to 2.6)	n/a[b]
Dabigatran 300+	2889	5.2 (0.0 to 13.7)	n/a[b]	421	1.3 (0.0 to 3.8)	n/a[b]
Rivaroxaban <20	3080	10.3 (5.9 to 14.6)	0.97 (0.66 to 1.45)	541	8.7 (0.5 to 16.9)	1.15 (0.49 to 2.68)
Rivaroxaban 20+	16771	4.1 (3.1 to 5.1)	0.63 (0.48 to 0.83)*	2303	4.9 (2.0 to 7.8)	0.85 (0.44 to 1.64)
Apixaban <10	3464	3.9 (1.5 to 6.4)	0.59 (0.38 to 0.93)	344	-	n/a[b]
Apixaban 10+	6863	2.8 (1.5 to 4.1)	0.43 (0.27 to 0.70)*	614	7.2 (0.0 to 15.1)	n/a[b]
**Haematuria**						
Dabigatran <300	3052	6.9 (3.6 to 10.3)	0.94 (0.62 to 1.43)	583	13.0 (0.0 to 27.4)	1.00 (0.41 to 2.44)
Dabigatran 300+	2878	7.6 (0.0 to 16.1)	0.76 (0.46 to 1.28)	421	1.3 (0.0 to 3.8)	n/a[b]
Rivaroxaban <20	3061	13.4 (8.1 to 18.7)	1.54 (1.09 to 2.19)	537	9.8 (3.0 to 16.6)	1.87 (0.89 to 3.90)
Rivaroxaban 20+	16704	7.7 (6.3 to 9.0)	1.04 (0.84 to 1.28)	2293	9.2 (5.2 to 13.3)	1.27 (0.76 to 2.13)
Apixaban <10	3459	7.1 (1.8 to 12.3)	0.88 (0.56 to 1.37)	343	17.6 (0.0 to 47.1)	n/a[b]
Apixaban 10+	6856	4.0 (2.4 to 5.5)	0.54 (0.36 to 0.81)*	613	6.4 (0.0 to 13.1)	n/a[b]
**Haemoptysis**						
Dabigatran <300	3081	1.5 (0.0 to 3.5)	n/a[b]	586	1.8 (0.0 to 5.4)	n/a[b]
Dabigatran 300+	2874	1.6 (0.4 to 2.8)	1.67 (0.77 to 3.62)	421	-	n/a[b]
Rivaroxaban <20	3079	1.6 (0.0 to 3.5)	1.10 (0.44 to 2.76)	541	6.5 (0.0 to 15.6)	n/a[b]
Rivaroxaban 20+	16767	1.6 (1.0 to 2.2)	1.05 (0.67 to 1.67)	2303	1.8 (0.0 to 3.6)	n/a[b]
Apixaban <10	3467	3.0 (0.0 to 7.2)	n/a[b]	344	1.3 (0.0 to 4.0)	n/a[b]
Apixaban 10+	6866	0.1 (0.0 to 0.3)	n/a[b]	614	-	n/a[b]
**All GI bleed**						
Dabigatran <300	3062	15.8 (9.5 to 22.2)	1.41 (1.06 to 1.87)	585	6.3 (1.0 to 11.6)	0.67 (0.30 to 1.52)

Dabigatran 300+	2873	11.3 (0.0 to 25.2)	0.90 (0.57 to 1.40)	419	51.7 (0.0 to 114.5)	1.48 (0.65 to 3.35)
Rivaroxaban <20	3069	11.2 (6.9 to 15.6)	1.12 (0.83 to 1.49)	540	16.1 (4.3 to 27.8)	0.99 (0.53 to 1.84)
Rivaroxaban 20+	16683	13.3 (11.5 to 15.1)	1.16 (0.98 to 1.37)	2292	15.6 (10.3 to 20.9)	0.94 (0.64 to 1.37)
Apixaban <10	3453	11.5 (4.8 to 18.2)	0.69 (0.49 to 0.97)	344	15.6 (0.0 to 36.2)	0.63 (0.25 to 1.56)
Apixaban 10+	6846	7.7 (5.5 to 10.0)	0.68 (0.51 to 0.92)	612	10.4 (1.8 to 19.1)	0.63 (0.28 to 1.45)
**Upper GI bleed**						
Dabigatran <300	3062	15.3 (9.0 to 21.6)	1.48 (1.11 to 1.98)*	585	6.3 (1.0 to 11.6)	0.75 (0.33 to 1.68)
Dabigatran 300+	2873	11.0 (0.0 to 24.9)	0.90 (0.56 to 1.44)	419	49.9 (0.0 to 112.6)	1.37 (0.56 to 3.34)
Rivaroxaban <20	3071	10.8 (6.5 to 15.1)	1.12 (0.83 to 1.51)	540	16.1 (4.3 to 27.8)	1.08 (0.58 to 2.01)
Rivaroxaban 20+	16693	12.4 (10.7 to 14.2)	1.17 (0.99 to 1.39)	2292	15.1 (9.9 to 20.2)	1.00 (0.67 to 1.48)
Apixaban <10	3453	10.6 (4.1 to 17.1)	0.67 (0.47 to 0.96)	344	15.6 (0.0 to 36.2)	0.68 (0.27 to 1.69)
Apixaban 10+	6849	7.4 (5.2 to 9.5)	0.70 (0.51 to 0.95)	614	8.9 (0.8 to 17.1)	0.58 (0.23 to 1.43)
**Rectal bleed**						
Dabigatran <300	3080	0.5 (0.0 to 1.3)	n/a[b]	587	-	n/a[b]
Dabigatran 300+	2889	0.6 (0.0 to 1.2)	n/a[b]	420	1.8 (0.0 to 5.3)	n/a[b]
Rivaroxaban <20	3081	0.4 (0.0 to 0.9)	n/a[b]	542	-	n/a[b]
Rivaroxaban 20+	16770	0.9 (0.4 to 1.3)	1.00 (0.55 to 1.83)	2303	0.5 (0.0 to 1.5)	n/a[b]
Apixaban <10	3467	1.1 (0.0 to 2.6)	n/a	344	-	n/a[b]
Apixaban 10+	6863	0.4 (0.0 to 0.8)	n/a	613	1.5 (0.0 to 4.3)	n/a[b]
**Ischaemic stroke[c]**						
Dabigatran <300	2095	16.0 (10.4 to 21.5)	1.18 (0.87 to 1.61)	385	15.6 (3.4 to 27.7)	1.10 (0.54 to 2.23)
Dabigatran 300+	2210	17.1 (4.5 to 29.7)	1.52 (1.07 to 2.15)	325	3.7 (0.0 to 8.9)	n/a[b]
Rivaroxaban <20	2338	20.4 (12.7 to 28.0)	1.11 (0.83 to 1.50)	397	21.1 (7.9 to 34.2)	1.40 (0.77 to 2.57)
Rivaroxaban 20+	14183	11.0 (9.2 to 12.8)	0.88 (0.73 to 1.06)	1886	15.5 (9.8 to 21.2)	1.08 (0.71 to 1.65)
Apixaban <10	2383	16.9 (9.5 to 24.4)	1.17 (0.87 to 1.57)	218	14.4 (0.0 to 30.5)	0.96 (0.38 to 2.39)
Apixaban 10+	5176	12.4 (9.4 to 15.5)	1.07 (0.82 to 1.40)	412	22.0 (4.1 to 39.9)	1.14 (0.52 to 2.49)
**VTE[d]**						
Dabigatran <300	2933	3.2 (0.6 to 5.9)	0.14 (0.07 to 0.28)*	550	6.8 (0.0 to 14.8)	n/a[b]
Dabigatran 300+	2799	1.5 (0.4 to 2.7)	0.25 (0.12 to 0.52)*	397	6.3 (0.0 to 13.7)	n/a[b]
Rivaroxaban <20	2761	17.9 (10.0 to 25.8)	0.24 (0.17 to 0.35)*	441	113.1 (67.3 to 158.9)	1.39 (0.91 to 2.12)
Rivaroxaban 20+	12506	55.6 (51.5 to 59.7)	1.92 (1.70 to 2.18)*	1728	54.0 (42.4 to 65.6)	2.04 (1.50 to 2.76)*
Apixaban <10	3234	11.5 (3.2 to 19.8)	0.26 (0.17 to 0.40)*	316	9.7 (0.0 to 21.8)	n/a[b]
Apixaban 10+	6328	14.8 (11.5 to 18.2)	0.60 (0.47 to 0.76)*	565	7.5 (0.0 to 15.3)	n/a[b]
**Mortality**						
Dabigatran <300	3081	53.1 (42.1 to 64.2)	1.12 (0.97 to 1.28)	587	59.2 (36.1 to 82.4)	1.14 (0.82 to 1.58)

Dabigatran 300+	2889	68.2 (20.8 to 115.6)	0.72 (0.55 to 0.95)	421	20.9 (8.6 to 33.3)	1.27 (0.72 to 2.25)
Rivaroxaban <20	3083	96.6 (83.4 to 109.8)	1.45 (1.30 to 1.62)*	542	126.6 (91.3 to 161.8)	1.78 (1.40 to 2.27)*
Rivaroxaban 20+	16780	61.5 (57.6 to 65.3)	1.24 (1.15 to 1.34)*	2304	60.1 (49.9 to 70.3)	1.23 (1.01 to 1.50)
Apixaban <10	3467	97.1 (81.1 to 113.2)	1.28 (1.15 to 1.42)*	344	105.9 (56.4 to 155.5)	1.63 (1.20 to 2.21)*
Apixaban 10+	6867	47.4 (41.8 to 53.0)	0.97 (0.86 to 1.10)	614	45.3 (27.1 to 63.5)	1.05 (0.70 to 1.57)

[a]) adjusted for age, sex, ethnicity, smoking, alcohol, Townsend quintile, BMI, SPB, falls&hipfracture, hip/knee ops, comorbidities (alcoholism, AF, treated hypertension, CKD, COPD, liver disease, CHD, CCF, any cancer, valvular, peptic ulcer), previous events (bleed, VTE, ischaemic stroke), medications at the baseline (macrolides, antiplatelets, anticonvulsant, corticosteroids, NSAIDs, statin, hormones), study year

[b]) insufficient data for analysis

[c]) based on patients without previous ischaemic stroke

[d]) based on patients without previous VTE

*) P-value<0.01

*eTable 14 **All patients**: Sensitivity analysis, censoring patients at the time of hospitalisation related to other outcome. Adjusted hazard ratios (95% CI) for outcomes associated with exposure to study drugs compared with warfarin.*

	QResearch		CPRD		Both databases	
Comparison with warfarin	Adjusted HR (95%CI)	P-value	Adjusted HR (95%CI)	P-value	Combined analysis HR (95%CI)	P-value
**Major bleeding**						
Dabigatran	0.92 (0.77 to 1.09)	0.3	0.75 (0.49 to 1.14)	0.2	0.89 (0.76 to 1.04)	0.1
Rivaroxaban	1.04 (0.94 to 1.15)	0.5	1.15 (0.92 to 1.45)	0.2	1.05 (0.96 to 1.16)	0.3
Apixaban	0.60 (0.51 to 0.71)	<0.001	0.76 (0.50 to 1.15)	0.2	0.62 (0.53 to 0.72)	<0.001
**Intracranial bleed**						
Dabigatran	0.43 (0.27 to 0.70)	<0.001	0.29 (0.07 to 1.17)	0.08	0.41 (0.26 to 0.65)	<0.001
Rivaroxaban	0.68 (0.54 to 0.87)	0.002	1.03 (0.61 to 1.74)	0.9	0.73 (0.58 to 0.91)	0.005
Apixaban	0.46 (0.32 to 0.66)	<0.001	0.58 (0.21 to 1.65)	0.3	0.47 (0.34 to 0.66)	<0.001
**Haematuria**						
Dabigatran	0.91 (0.66 to 1.25)	0.6	0.92 (0.45 to 1.88)	0.8	0.91 (0.68 to 1.21)	0.5
Rivaroxaban	1.15 (0.95 to 1.39)	0.2	1.52 (0.99 to 2.34)	0.06	1.20 (1.00 to 1.42)	0.05
Apixaban	0.67 (0.49 to 0.91)	0.01	1.18 (0.57 to 2.44)	0.7	0.73 (0.55 to 0.97)	0.03
**Haemoptysis**						
Dabigatran	1.23 (0.64 to 2.36)	0.5	0.75 (0.10 to 5.58)	0.8	1.17 (0.63 to 2.17)	0.6
Rivaroxaban	1.14 (0.74 to 1.74)	0.6	1.46 (0.53 to 3.99)	0.5	1.17 (0.79 to 1.72)	0.4
Apixaban	0.33 (0.13 to 0.85)	0.02	0.95 (0.12 to 7.60)	1	0.39 (0.17 to 0.92)	0.03
**All GI bleed**						
Dabigatran	1.18 (0.93 to 1.51)	0.2	0.88 (0.49 to 1.58)	0.7	1.13 (0.90 to 1.42)	0.3
Rivaroxaban	1.16 (1.00 to 1.35)	0.06	1.01 (0.72 to 1.40)	1	1.13 (0.99 to 1.30)	0.07
Apixaban	0.67 (0.53 to 0.85)	0.001	0.62 (0.33 to 1.17)	0.1	0.66 (0.53 to 0.83)	<0.001
**Upper GI bleed**						
Dabigatran	1.21 (0.94 to 1.56)	0.1	0.90 (0.49 to 1.66)	0.7	1.16 (0.91 to 1.46)	0.2
Rivaroxaban	1.18 (1.01 to 1.38)	0.04	1.08 (0.77 to 1.52)	0.6	1.17 (1.01 to 1.35)	0.03
Apixaban	0.67 (0.52 to 0.85)	0.001	0.62 (0.32 to 1.20)	0.2	0.66 (0.52 to 0.83)	<0.001
**Rectal bleed**						
Dabigatran	0.98 (0.39 to 2.42)	1	0.70 (0.09 to 5.26)	0.7	0.92 (0.40 to 2.11)	0.8
Rivaroxaban	0.95 (0.53 to 1.73)	0.9	0.23 (0.03 to 1.81)	0.2	0.86 (0.48 to 1.51)	0.6
Apixaban	0.87 (0.37 to 2.04)	0.7	0.64 (0.08 to 5.26)	0.7	0.82 (0.37 to 1.82)	0.6
**Ischaemic stroke**						
Dabigatran	1.31 (1.03 to 1.66)	0.03	0.89 (0.47 to 1.69)	0.7	1.25 (1.00 to 1.56)	0.05
Rivaroxaban	0.94 (0.79 to 1.11)	0.5	1.04 (0.71 to 1.53)	0.8	0.97 (0.83 to 1.13)	0.7
Apixaban	1.16 (0.94 to 1.43)	0.2	1.02 (0.55 to 1.90)	0.9	1.14 (0.94 to 1.39)	0.2
**VTE**						
Dabigatran	0.20 (0.12 to 0.34)	<0.001	0.50 (0.23 to 1.07)	0.07	0.26 (0.17 to 0.40)	<0.001
Rivaroxaban	1.50 (1.33 to 1.70)	<0.001	1.88 (1.42 to 2.48)	<0.001	1.55 (1.39 to 1.74)	<0.001
Apixaban	0.46 (0.37 to 0.57)	<0.001	0.50 (0.24 to 1.03)	0.06	0.46 (0.37 to 0.57)	<0.001
**Mortality**						
Dabigatran	1.02 (0.90 to 1.16)	0.7	1.16 (0.87 to 1.53)	0.3	1.04 (0.92 to 1.16)	0.5
Rivaroxaban	1.34 (1.25 to 1.43)	<0.001	1.46 (1.24 to 1.71)	<0.001	1.36 (1.27 to 1.44)	<0.001
Apixaban	1.13 (1.03 to 1.24)	0.009	1.36 (1.06 to 1.76)	0.02	1.16 (1.06 to 1.26)	<0.001

*) adjusted for age, sex, ethnicity, smoking, alcohol, Townsend quintile, BMI, SPB, falls&hipfracture, hip/knee ops, comorbidities (alcoholism, AF, treated hypertension, CKD, COPD, liver disease, CHD, CCF, any cancer, valvular, peptic ulcer), previous events (bleed, VTE, ischaemic stroke), medications at the baseline (macrolides, antiplatelets, anticonvulsant, corticosteroids, NSAIDs, statin, hormones), study year

*eTable 15 **All patients**: Complete case analysis and propensity score adjustment by database. Adjusted hazard ratios (95% CI) for outcomes associated with exposure to study drugs compared with warfarin.*

	QResearch		CPRD	
Comparison with warfarin	Complete case analysis* (N=141,760)	Propensity score adjustment#	Complete case analysis* (N=36,465)	Propensity score adjustment†
**Major bleeding**				
Dabigatran	0.93 (0.78 to 1.11)	0.87 (0.73 to 1.04)	0.79 (0.52 to 1.22)	0.72 (0.47 to 1.12)
Rivaroxaban	1.03 (0.92 to 1.14)	0.97 (0.88 to 1.06)	1.15 (0.90 to 1.46)	1.03 (0.83 to 1.28)
Apixaban	0.60 (0.51 to 0.71)	0.60 (0.51 to 0.70)	0.74 (0.47 to 1.14)	0.71 (0.47 to 1.07)
**Intracranial bleed**				
Dabigatran	0.46 (0.28 to 0.75)	0.50 (0.31 to 0.80)	0.31 (0.08 to 1.25)	0.24 (0.04 to 1.43)
Rivaroxaban	0.66 (0.51 to 0.85)	0.67 (0.53 to 0.85)	0.96 (0.55 to 1.67)	0.89 (0.54 to 1.48)
Apixaban	0.42 (0.29 to 0.62)	0.51 (0.36 to 0.72)	0.59 (0.21 to 1.66)	0.52 (0.19 to 1.43)
**Haematuria**				
Dabigatran	0.89 (0.64 to 1.25)	0.78 (0.56 to 1.08)	1.06 (0.51 to 2.16)	0.87 (0.42 to 1.81)
Rivaroxaban	1.17 (0.96 to 1.42)	0.94 (0.79 to 1.12)	1.56 (0.98 to 2.47)	0.89 (0.58 to 1.38)
Apixaban	0.67 (0.48 to 0.92)	0.51 (0.38 to 0.69)	1.06 (0.47 to 2.38)	0.71 (0.33 to 1.53)
**Haemoptysis**				
Dabigatran	1.24 (0.65 to 2.39)	1.09 (0.56 to 2.13)	0.88 (0.12 to 6.56)	0.59 (0.08 to 4.42)
Rivaroxaban	0.97 (0.62 to 1.52)	0.88 (0.58 to 1.33)	1.29 (0.44 to 3.76)	0.89 (0.31 to 2.57)
Apixaban	0.34 (0.13 to 0.88)	0.30 (0.12 to 0.73)	1.01 (0.13 to 8.14)	0.65 (0.08 to 5.05)
**All GI bleed**				
Dabigatran	1.23 (0.96 to 1.58)	1.14 (0.89 to 1.45)	0.87 (0.47 to 1.60)	0.80 (0.42 to 1.52)
Rivaroxaban	1.17 (1.00 to 1.37)	1.19 (1.03 to 1.37)	1.01 (0.72 to 1.43)	1.14 (0.84 to 1.55)
Apixaban	0.71 (0.56 to 0.91)	0.77 (0.62 to 0.96)	0.63 (0.33 to 1.18)	0.75 (0.41 to 1.37)
**Upper GI bleed**				
Dabigatran	1.27 (0.98 to 1.65)	1.20 (0.93 to 1.55)	0.87 (0.46 to 1.64)	0.82 (0.42 to 1.57)
Rivaroxaban	1.18 (1.00 to 1.39)	1.24 (1.07 to 1.44)	1.08 (0.76 to 1.53)	1.23 (0.90 to 1.68)
Apixaban	0.72 (0.56 to 0.92)	0.81 (0.64 to 1.02)	0.61 (0.31 to 1.18)	0.74 (0.40 to 1.40)
**Rectal bleed**				
Dabigatran	0.91 (0.37 to 2.26)	0.69 (0.28 to 1.70)	0.88 (0.12 to 6.68)	0.13 (0.00 to 10.41)
Rivaroxaban	1.02 (0.58 to 1.78)	0.72 (0.43 to 1.19)	0.27 (0.03 to 2.17)	0.23 (0.03 to 1.70)
Apixaban	0.79 (0.34 to 1.85)	0.50 (0.23 to 1.09)	0.81 (0.10 to 6.79)	0.71 (0.09 to 5.38)
**Ischaemic stroke**				
Dabigatran	1.29 (1.01 to 1.65)	1.32 (1.04 to 1.68)	0.76 (0.38 to 1.56)	0.65 (0.25 to 1.65)
Rivaroxaban	0.93 (0.79 to 1.11)	0.85 (0.72 to 0.99)	1.07 (0.72 to 1.58)	1.02 (0.72 to 1.44)
Apixaban	1.16 (0.93 to 1.44)	1.10 (0.91 to 1.33)	0.98 (0.52 to 1.88)	1.02 (0.55 to 1.89)
**VTE**				
Dabigatran	0.20 (0.12 to 0.34)	0.21 (0.12 to 0.36)	0.48 (0.21 to 1.10)	0.45 (0.16 to 1.32)
Rivaroxaban	1.51 (1.32 to 1.72)	2.41 (2.17 to 2.67)	1.85 (1.37 to 2.52)	2.26 (1.70 to 3.00)
Apixaban	0.44 (0.35 to 0.56)	0. 59 (0.47 to 0.73)	0.43 (0.19 to 0.99)	0.38 (0.16 to 0.89)
**Mortality**				
Dabigatran	1.00 (0.88 to 1.14)	0.93 (0.82 to 1.06)	1.02 (0.74 to 1.39)	0.93 (0.68 to 1.27)
Rivaroxaban	1.30 (1.22 to 1.40)	1.37 (1.29 to 1.46)	1.44 (1.22 to 1.70)	1.59 (1.38 to 1.84)
Apixaban	1.12 (1.02 to 1.23)	1.34 (1.24 to 1.46)	1.40 (1.08 to 1.81)	1.52 (1.20 to 1.93)

*) adjusted for age, sex, ethnicity, smoking, alcohol, Townsend quintile, BMI, SPB, falls&hipfracture, hip/knee ops, comorbidities (alcoholism, AF, treated hypertension, CKD, COPD, liver disease, CHD, CCF, any cancer, valvular, peptic ulcer), previous events (bleed, VTE, ischaemic stroke), medications at the baseline (macrolides, antiplatelets, anticonvulsant, corticosteroids, NSAIDs, statin, hormones), study year

#) based on 5,832 dabigatran users compared to 91,917 warfarin users; 28,652 rivaroxaban users compared to 87,108 warfarin users; 15,012 apixaban users compared to 86,233 warfarin users.

†) based on 1,144 dabigatran users compared to 26,693 warfarin users; 4,981 rivaroxaban users compared to 24,607 warfarin users; 1,664 apixaban users compared to 21,824 warfarin users.
