# Supplementary material for: Risks and benefits of direct oral anticoagulants versus warfarin in a real world setting: cohort study in primary care
Source: BMJ. 2018 Jul 4;362:k2505. doi: 10.1136/bmj.k2505 (PMC6031213; doi:10.1136/bmj.k2505)
Supplement: Supplementary file 2 — Supplementary information: Supplementary figure 1 [file viny041857.ww2.pdf]

eFigure 1: **All patients**: Adjusted Cox hazard ratios (95% CI) for outcomes associated with exposure to study drugs overall and by prescribed dose compared with warfarin

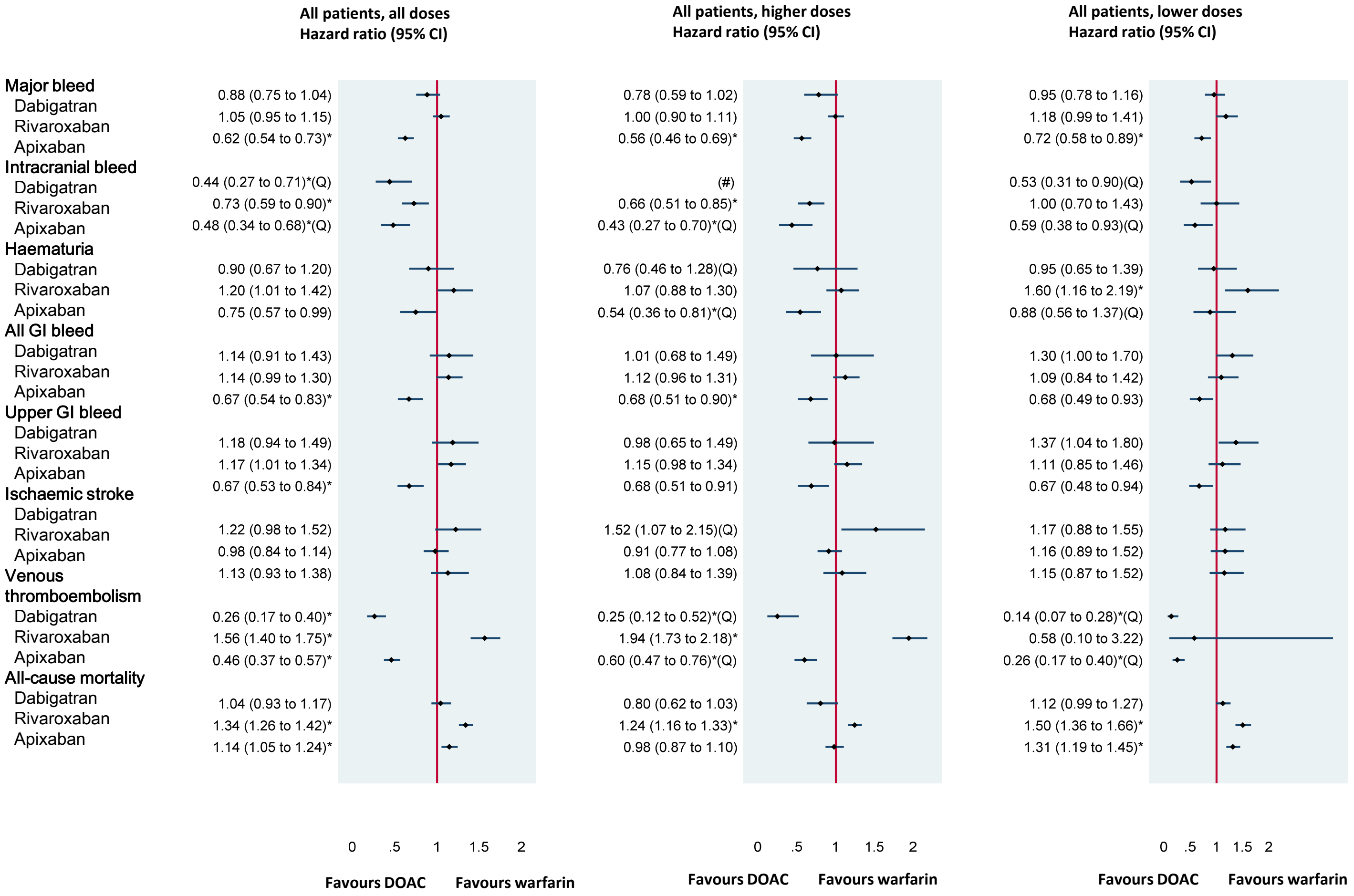

Hazard ratios are adjusted for age, sex, ethnicity, smoking, alcohol, Townsend quintile, BMI, SPB, falls&hipfracture, hip/knee ops, comorbidities (alcoholism, treated hypertension, CKD, COPD, liver disease, CHD, CCF, any cancer, valvular, peptic ulcer), previous events (bleed, VTE, ischaemic stroke), medications at the baseline (macrolides, antiplatelets, anticonvulsant, corticosteroids, NSAIDs, statin, hormones), study year

*) P-value<0.01

Q) The results were only available from QResearch analysis
